# Evidence for Endogenous Collagen in *Edmontosaurus* Fossil Bone

**DOI:** 10.1021/acs.analchem.4c03115

**Published:** 2025-01-17

**Authors:** Lucien Tuinstra, Brian Thomas, Steven Robinson, Krzysztof Pawlak, Gazmend Elezi, Kym Francis Faull, Stephen Taylor

**Affiliations:** 1 Department of Electrical Engineering and Electronics, University of Liverpool, Liverpool L69 3BX, U.K.; 2 Materials Innovation Factory, University of Liverpool, Liverpool L7 3NY, U.K.; 3 Pasarow Mass Spectrometry Laboratory, Jane and Terry Semel Institute for Neuroscience and Human Behaviour and Department of Psychiatry & Biobehavioral Sciences, David Geffen School of Medicine, University of California, Los Angeles 90095, United States; 4 Former Director of Pasarow Mass Spectrometry Laboratory, Jane and Terry Semel Institute for Neuroscience and Human Behaviour and Department of Psychiatry & Biobehavioral Sciences, David Geffen School of Medicine, University of California, Los Angeles 90095, United States

## Abstract

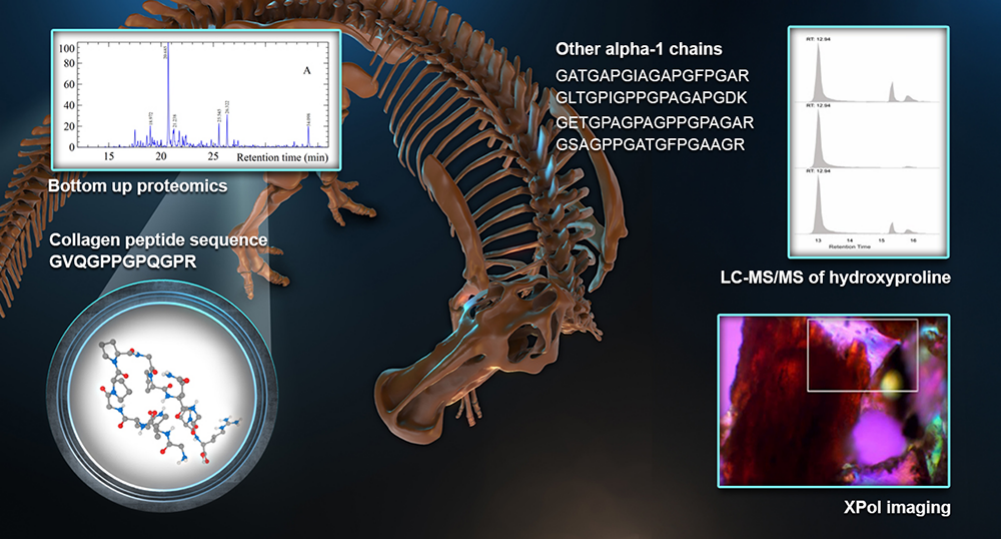

Reports of proteins in fossilized bones have been a subject of controversy in the scientific literature because it is assumed that fossilization results in the destruction of all organic components. In this paper, a novel combination of analytical techniques is used to address this question for an exceptionally well-preserved *Edmontosaurus* sacrum excavated from the Upper Cretaceous strata of the South Dakota Hell Creek Formation. Cross-polarized light microscopy (XPol) shows birefringence consistent with collagen presence. Tandem LC-MS unambiguously identified, and for the first time quantified, hydroxyproline, a unique collagen-indicator amino acid, in acid-digested samples from the *Edmontosaurus.* LC-MS/MS bottom-up proteomics shows identical collagen peptide sequences previously identified and reported for another hadrosaur and a *T. rex* sample.

## Introduction

Bone stability and the temporal decay of organic molecules is of interest in palaeontology,^1,2^ archeology,^3^ and forensics.^4^ The state of decay can provide information regarding burial conditions, e.g. aerobic/anaerobic etc.^5^ and disease status.^6^

The bones of all vertebrate animals contain proteins including collagen which decay as a consequence of bio- and environmentally induced degradation *post mortem*.^7−9^ In large animals, due to the bone size and initially high protein abundance, with modern techniques it is possible to identify and quantify protein remnants in ancient samples. A review of soft tissue preservation in palaeontological samples from different strata and locations reveals widespread occurrence (see Thomas and Taylor and references therein^10^).

Using scanning electron microscopy (SEM), Pawlicki et al. in 1966 reported collagenous material in the phalange bone of a dinosaur from the Upper Cretaceous.^11^ In 1999 collagen fibers were reported in *T. rex* bone (Museum of the Rockies MOR 555) from the Hell Creek Formation using transmission electron microscopy (TEM).^12^ Attempts to identify residual hemoglobin and heme were inconclusive and this remains an active research area.^13^ The examination of another *T. rex* bone (MOR 1125) from the same formation using SEM revealed tissue flexibility which was unanticipated.^14^ Secondary ion mass spectrometry (SIMS) was later used and protein endogeneity was proposed.^15^

In 2008, multiple layers of collagenous fibers were reported in *Psittacosaurus* skin from the Lower Cretaceous Xixian Formation.^16^ Sauropodomorph embryos from the Lower Jurassic were assessed using synchrotron radiation Fourier transform infrared spectroscopy (SR-FTIR) which indicated the presence of amide and apatite peaks within woven embryonic bone tissue.^17^ Another study used FTIR, Raman and second harmonic generation (SHG) to confirm collagen in samples of modern, medieval, and ice-age bones.^18^

Histochemical and immunological evidence was concluded to support collagen type II presence in *Hypacrosaurus stebingeri*, from a duck-billed dinosaur (MOR 548) from the Upper Cretaceous. The authors argue that microbial contamination could be eliminated as the protein source, since microbes are incapable of producing collagen. Intercalating DNA staining was observed and the survival of endogenous nuclear material was suggested.^19^

Studies using Mass Spectrometry (MS) include Asara et al.^20^ They sequenced collagen fragments from a mastodon (MOR 605) and *T.Rex* (MOR 1125) using liquid chromatography tandem mass spectrometry (LC-MS/MS), concluding long-term stability of peptide bonds. This was followed in 2009 by time-of-flight (ToF)-secondary ion mass spectrometry (SIMS) study of *Brachylophosaurus canadensis* (MOR 2598) fossils.^21^ Hydroxyproline (Hyp, C_5_H_9_NO_3_) was identified, a relatively rare amino acid but abundant in collagen. Further study on the same bone confirmed earlier findings and a further six collagen I peptides were sequenced.^22^ In 2009, a study of *Edmontosaurus* (*sp.*) using FTIR suggested the presence of amide-containing compounds (absorption peaks around 1650 cm^–1^) and pyrolysis gas chromatography (GC)-MS confirmed endogenous organics.^23^ Lee et al. published their evidence of preserved collagen I in a Jurassic sauropod *Lufengosaurus* using SR-FTIR.^24^ Using the same technique, Boatman et al. also showed strong amide I and amide II absorption bands in *T. rex* vessels, consistent with collagen presence. Scanning electron microscope (SEM) imaging showed a triple helix (consistent with fibrillar collagen).^25^

The above authors report preservation of original collagen over long time periods, detected by an array of techniques. However, the endogeneity of protein remnants in paleontological bones has been contested with some maintaining that all original (endogenous) proteins should long ago have been replaced by the process of mineralization and can no longer be found *in situ*.^26−29^

In this paper we use attenuated total reflectance (ATR)-FTIR^30,31^ and cross-polarized light microscopy (XPol)^32^ supplemented by two MS techniques to elucidate the question of collagen endogeneity in *Edmontosaurus sp.* fossil bone (UOL GEO.1). LC-MS/MS is used to identify hydroxyproline and enzymatic digestion followed by MS to yield partial amino acid sequences which are used in database searching to identify specific proteins.^33^

## Methods

### Samples and Preparation

Herbivorous *Edmontosaurus sp.* (Hadrosauridae) sacrum bone fossils were excavated from the Upper Cretaceous zone of the Hell Creek Formation in Harding County, South Dakota, USA (45°.56″N, −103°.46″W) in 2019. A 20 kg sample from this duck-billed dinosaur fossil together with samples of the accompanying sediment was donated to, and accessioned at the repository of the Victoria Gallery & Museum of the University of Liverpool under UOL GEO.1.

Motion photogrammetry was used to capture a digital 3D model of the *Edmontosaurus sp.* bone fossils prior to analysis (see Supplementary Table S1).

For comparison and control a modern bone from a common turkey (*Meleagris gallopavo*), sourced from a local butcher and because it is often classed in the *Archosauria,* and pure Bovine tendon collagen (Sigma-Aldrich product #5162) were used. Small bone segments (in the order of a few grams) were dried in an oven at 60 °C for several hours in preparation for crushing (powderisation). The same analysis protocols were used for both samples. The samples were ground bone shards (cross sections of 1–3 mm thick, Figure 2) prepared using a mortar and pestle. The shards were cleaned with powdered bicarbonate and hot water (∼50 °C) before final rinsing with deionized water. The samples were ground to powder with particle sizes of no more than 50 μm [40]. A 50-μm stackable zooplankton sieve was used to filter the particles onto a freshly cut piece of aluminum foil for transfer into new vials, ready for LC-MS/MS analysis.

### FTIR

FTIR was performed using an Attenuated Total Reflectance accessory (ATR) with a germanium window on a Bruker Vertex 70© equipped with a Deuterated Lanthanum α Alanine doped TriGlycine Sulfate (DLaTGS) detector. Each spectrum combined an average of 32 scans, with a resolution of between 2–4 cm^–1^ in the range of 4500 to 650 cm^–1^. Spectra were collected and analyzed with OPUS software and compared with authentic Ca_3_PO_4_ from the library (©Nicodom, 2014). Absorption maxima correspond to the moiety abundance in the sample absorbing the energy at a certain frequency.

### XPol

Thin sections of UOL GEO.1 were prepared according to Chinsamy and Raath.^34^ Accordingly, polyvinyl acetate was used as the binding agent and applied to the bone-glass contact surface only. Thin sections were polished to thickness of 16 μm and imaged using a Motic Polarizing Microscope BA310 with a Sony ILCE-7RM4 detector. Images from several focal planes were collected then stacked using Photoshop 24.5.

### LC-MS/MS Bottom-Up Proteomics

Twenty milligrams each of *Edmontosaurus* bone, turkey bone, and bovine collagen was dispensed into separate polypropylene microcentrifuge tubes. Each sample was treated with aqueous ammonium bicarbonate (AmBic, 80 μL, 25 mM) and RapiGest SF Surfactant solution (1% RapiGest solution in AmBic, 5 μL, Waters) with continuous gentle shaking (450 rpm, 80 °C, 10 min.). Cysteine reduction was then performed by the addition of dithiothreitol (DTT, 11.1 mg/mL in 25 mM AmBic, 5 μL). After mixing and incubation (60 °C, 10 min.) alkylation of free thiols was performed using iodoacetamide (46.6 mg/mL in 25 mM Ambic, 5 μL, 30 min in the dark). Excess iodoacetamide was quenched with DTT (4.7 μL as above), and samples were acidified (neat trifluoroacetic acid, 2 μL) to a pH of 2 or less (checked with pH indicator paper). Digestion was carried out with trypsin (Promega sequencing grade, 0.2 μg/μL in 50 mM aqueous acetic acid) with incubation (37 °C, 16 h). Following centrifugation (13,000*g*, 15 min, 4 °C) the supernatants were transferred to clean microcentrifuge tubes and stored frozen until analysis.

Samples were analyzed using nanobore reversed-phase chromatography (Ultimate 3000 RSLC, Thermo Scientific, Hemel Hempstead) coupled to a hybrid linear quadrupole/orbitrap mass spectrometer (Q Exactive HF Quadrupole-Orbitrap, Thermo Scientific) equipped with a nanospray ionization source. Samples (2 μL) were loaded onto the trapping column (Thermo Scientific, PepMap100, C18, 300 μm x 5 mm) equilibrated in aqueous formic acid (0.1%, v/v) using partial loop injection over 7 min at a flow rate of 12 μL/min. After the direction of eluent flow was reversed components were transferred to and resolved on an analytical column (Easy-Spray C18 75 μm x 500 mm, 2 μm particle size) equilibrated in 96.2% eluent A (water/formic acid, 100/0.1, v/v) and 3.8% eluent B (acetonitrile/water/formic acid, 79.95/19.95/0.1, v/v/v) and eluted (0.3 μL/min) with a linear increasing concentration of eluant B (min/% B; 0/3.8, 30/50). The mass spectrometer was operated in a data-dependent positive ion mode (fwhm 60,000 orbitrap full-scan, automatic gain control (AGC) set to 3e^6^ ions, maximum fill time (MFT) of 100 ms). The seven most abundant peaks per full scan were selected for high energy collisional dissociation (HCD, 30,000 fwhm resolution, AGC 1e^5^, MFT 300 ms) with an ion selection window of 2 *m*/*z* and a normalized collision energy of 30%. Ion selection excluded singularly charged ions and ions with ≥ +6 charge state. A 60 s dynamic exclusion window was used to avoid repeated selection of the same ion for fragmentation.

Survey analyses of each sample were first used to determine the sample amount, calculated by extrapolation, needed to give a full orbitrap scan base peak intensity (BPI) of 1–2 x10^9^. These analyses were performed with a compacted 15 min gradient (Supplementary Table S2). Based on these BPI results, 2 μL of neat *Edmontosaurus* sample was used for the full analysis. The modern turkey sample was diluted 1:100 and Bovine collagen sample was diluted 1:1000 in water/acetonitrile/trifluoroacetic acid (97/3/0.1, v/v/v).

Typically, one or two blanks would be run once finishing test runs. Here, four 30 min blank analyses (injection solvent only) were performed after the turkey and bovine samples to minimize carry over, then the fossilized sample was analyzed on the 1 h program. The blank (water/acetonitrile/formic acid, 97/3/0.1, v/v/v) was resolved on the analytical column (Easy-Spray C18 75 μm x 500 mm 2 μm particle size) equilibrated in 96.2% eluent A (water/formic acid, 100/0.1, v/v) and 3.8% eluent B (acetonitrile/water/formic acid, 79.95/19.95/0.1, v/v/v) and eluted (0.3 μL/min.) with a linear increasing concentration of eluant B (min/% B; 0/3.8, 15/50).

### Database Searches

The data files were imported into PEAKS 11 (Bioinformatics Solutions Inc.) for searching the reviewed SwissProt database (569516 sequences), as well as the mixed—reviewed/unreviewed, one gene-one protein—UniCow (23841 sequences), UniTurkey (16212 sequences) and UniChick (18369 sequences) databases (all downloaded 05–04–23). The search parameters included cysteine carbamidomethylation, methionine oxidation, variable lysine and proline oxidation, a precursor mass tolerance of 10 ppm, a product mass tolerance of 0.01 Da, and a maximum of one missed cleavage. This software permits database searching for multiple post translation modifications (PTMs). The *Edmontosaurus* sample was searched against the SwissProt database, bovine collagen (96%) was searched against UniCow, and the modern turkey sample was searched against both UniChick and UniTurkey databases. The contaminants (cRAP) database was also included in each search.^35^

### LC-MS/MS of Hydroxyproline

Bone samples from the *Edmontosaurus* and modern turkey, and pure bovine collagen samples, were simultaneously processed and analyzed. After being frozen with liquid nitrogen, both fossilized and turkey bone samples were manually crushed to a fine powder with a mortar and pestle. One-gram portions of the powdered bone and 5 mg of bovine pure collagen were dispensed into polypropylene microcentrifuge tubes, suspended in water (1 mL), mixed vigorously, sonicated in a bath sonicator (30 min.), centrifuged (2000g, 15 min.), and the supernatants transferred to new tubes. The extraction procedure was repeated on the pellet by adding methanol (1 mL) and the samples were mixed, sonicated, and centrifuged as above. The supernatants were pooled and reserved for future bottom-up proteomics. The pellets were then treated with HCl (2 mL, 6 N) and incubated (2 h, 60 °C) before the samples were dried in a vacuum centrifuge. The HCl treatment was repeated until the samples ceased effervescing after HCl addition, each time with drying in a vacuum centrifuge between acid treatments. The repeated HCl treatments are necessary to remove all carbonate from the samples prior to attempting protein hydrolysis. Residual carbonate would completely or partially neutralize the acid necessary for amide bond cleavage. Removal of all carbonate was judged to be complete when there was no effervescence of the samples after the addition of acid, and was checked with pH paper indicator to ensure the samples were strongly acidic before proceeding with the protein hydrolysis treatment. Generally, it took two or three such treatments before effervescing ceased. The final dried samples were treated again with HCl (500 μL, 6 N) and incubated (12 h, 120 °C) to effect protein hydrolysis. The samples were dried overnight in a vacuum centrifuge and then treated with n-butanolic HCl (300 μL, 3 N), incubated (2 h, 60 °C) to make the butyl esters, and dried again in a vacuum centrifuge. Lastly, the samples were reconstituted in water (200 μL), mixed vigorously, and centrifuged (5 min, 16,000g, room temperature). The supernatants were transferred to HPLC vials and aliquots (typically 10 μL), injected onto a reversed-phase HPLC column (Phenomenex Kinetex, 2.6 μm Polar C18, 100 Å, 100 × 2.1 mm), equilibrated in eluant A, and eluted (100 μL/min) with a stepwise linearly increasing concentration of eluant C (acetonitrile/formic acid, 100/0.1, v/v; min/%C, 0/1, 5/1, 20/25, 22/1, 60/1). The effluent from the column was passed through an electrospray ionization (ESI) source (spray voltage 4.5 kV) connected to a hybrid linear ion trap/orbitrap mass spectrometer (Thermo Scientific Orbitrap LTQ XL) scanning in the positive ion mode. For the collection of ion trap mass spectra, the following instrument parameters were used: sheath gas flow rate 30 (arbitrary units), auxiliary gas flow rate 5 (arbitrary units), capillary temperature 300 °C, spray voltage 4,500 V, capillary voltage 22 V, tube lens voltage 110 V. For the collection of orbitrap mass spectra (accurate *m*/*z* measurements) immediately after calibration with LTQ ESI Positive Ion calibration solution mix, the same ESI settings were used with the following mass spectrometer parameters: mass range normal, fwhm resolution 100,000, scan range 50–1000 *m*/*z*. For the collection of ion trap fragment ion spectra of the butyl ester of hydroxyproline (Hyp_be_, MH^+^ at *m*/*z* 188), the following instrument parameters were used: mass range normal, scan range 50–200 *m*/*z*, collision energy 35, activation time 30 ms. Data were collected and interrogated with instrument manufacturer-supplied software (Xcalibur 2.05).

Control samples were included with each batch of bone samples. These were negative control samples devoid of added bone extracts (in triplicate), bovine collagen (20 mg/sample, in triplicate), and authentic Hyp standard in a range of amounts (typically 0, 2, 10, 20, and 50 nmol/sample, in duplicate). These samples were prepared and processed with each batch of bone samples. The order in which samples were analyzed was carefully arranged. Injections of water (solvent blanks) were used at the start of the analysis of each batch of samples to check that there were no peaks for Hyp_be_ resulting from carry-over from the analysis of previous sample batches. After verification of LC/MS system cleanliness, a typical order of sample analysis was: negative control samples 1–3; water blank #1; *Edmontosaurus* fossilized bone samples 1–3, water blanks #2 and #3, turkey bone samples 1–3, water blanks #4 and #5, collagen samples 1–3, water blanks #6 and #7, Hyp_be_ standards 1–10, water blanks #8–#10.

The data from the standard Hyp_be_ samples were processed by plotting the known amount of Hyp per sample against the measured chromatographic peak areas corresponding to the Hyp_be_ peak. The trendline equation was then used to interpolate or extrapolate the amount of Hyp in each sample.

## Results

**Figure 1:**
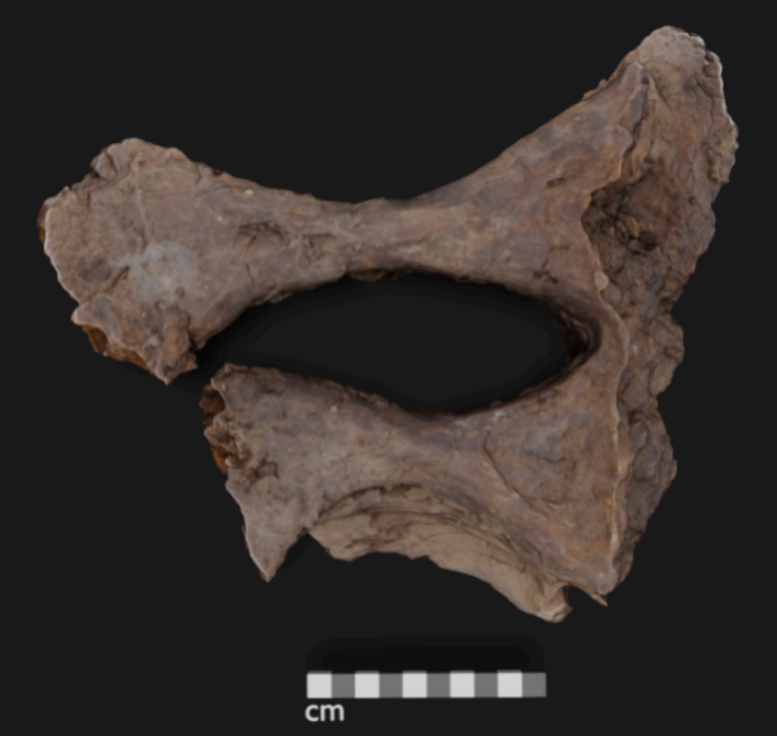
*Edmontosaurus sp.* sacrum (UOL GEO.1) from Harding County, SD, Hell Creek formation, main fragment shown.

**Figure 2:**
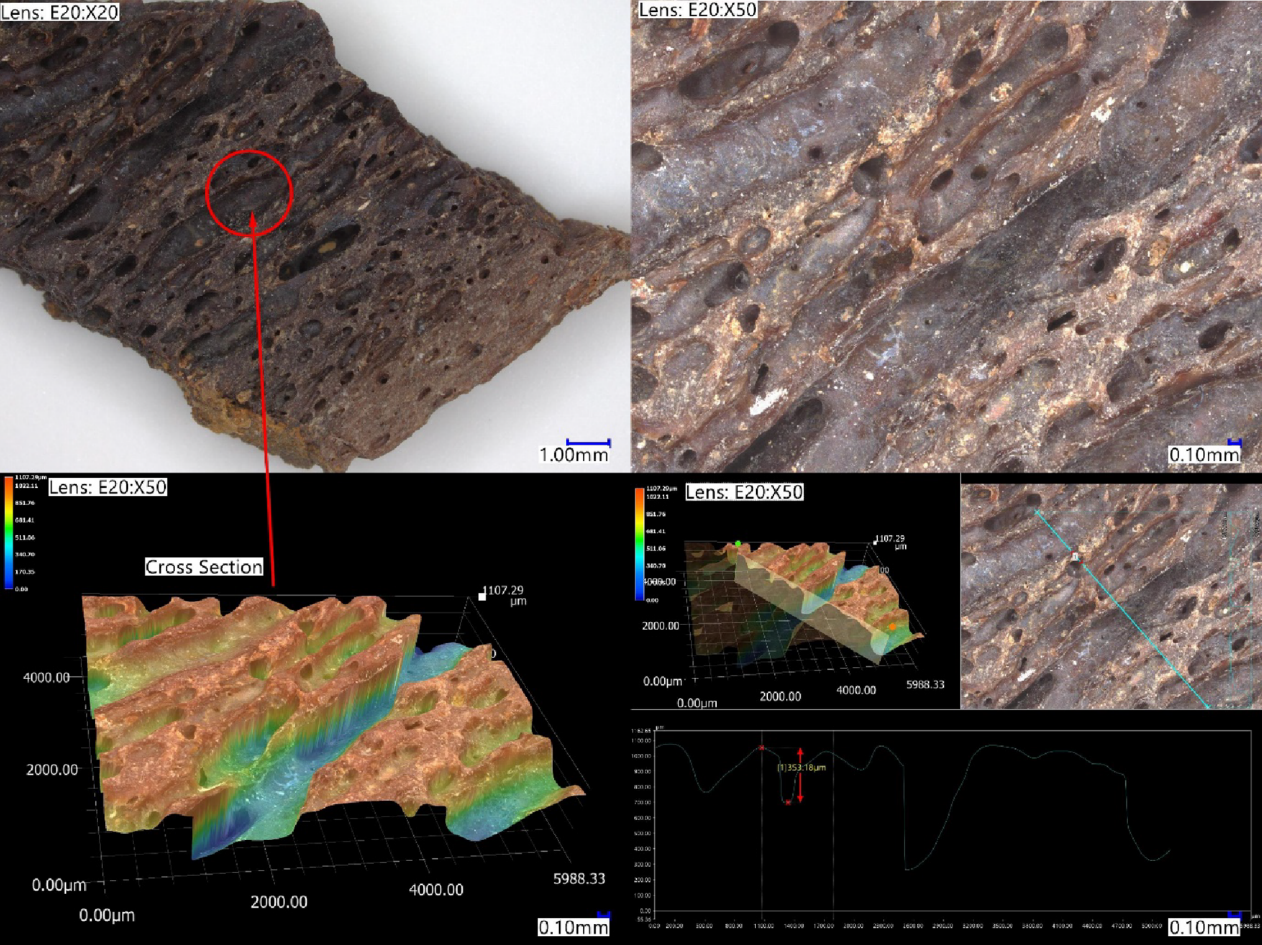
*Edmontosaurus* sections imaged using high resolution digital microscopy (Keyence VHX-7000).

After initial cleaning the fossilized *Edmontosaurus sp.* sacrum (UOL GE0.1) bone material weighed in total approximately 20 kg and the main fragment was intact (Figure 1), needing little stabilizing. The 3D model (Supplementary Table S1) allows detailed inspection of the surface topography of the bone aiding surface identification of postdepositional breaks and geometric measurements. Photogrammetric analysis showed that UOL GEO.1 had residual integrity. Trabecular bone is visible to the eye and also by digital microscopy (Figure 2).

### FTIR

**Figure 3:**
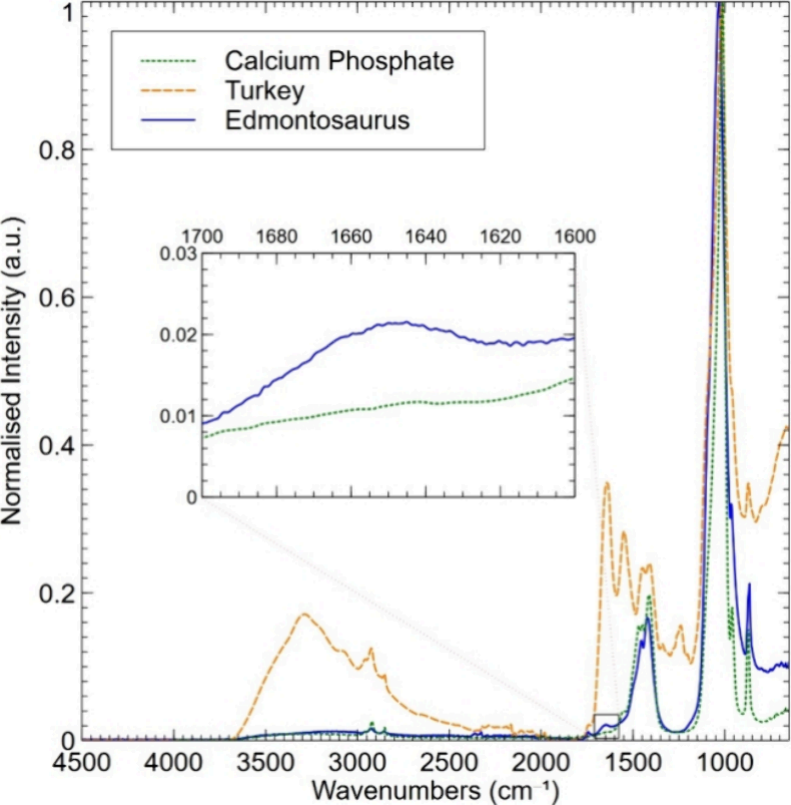
FTIR spectra from wavenumbers 4500 to 650 cm^–1^ of *Edmontosaurus* (solid blue line) and *Meleagris* (turkey, dashed orange line) bone overlaid with that obtained from calcium phosphate (dotted green line). The band around wavenumber 1033 cm^–1^ is assigned as indicating the presence of inorganic phosphate (PO_4_) in all samples. The inset shows an expanded presentation of the *Edmontosaurus* and calcium phosphate data around wavenumber 1650 cm^–1^ revealing an absorption maximum in the dinosaur spectrum consistent with the presence of a carbonyl moiety. (Graph generated using Veusz 3.6.2 software).

The inorganic component of the assemblage of bone is composed of hydroxyapatite (bioapatite).^31^ Antisymmetric stretching of PO_4_ occurs between approximate wavenumbers 1000–1100 cm^–1^, depending on the dipole change of the moiety.^36,37^ The FTIR spectra recorded for *Edmontosaurus* fossilized bone, turkey bone, and inorganic calcium phosphate all show a strong absorption around wavenumber 1050 cm^–1^ (Figure 3). The organic component of fresh bones comprises mostly type I collagen. An FTIR spectrum of collagen will show a band for amide I group (containing carbonyl, C = O) absorption around 1650 cm^–1^.^25,32^ The band visible around 1652 cm^–1^ in the modern turkey bone (Figure 3) likely indicates the presence of collagenous protein.^25^ As expected, this absorption maximum is not evident in the spectrum obtained from calcium phosphate. However, neither is this amide I (nor amide II) band present in the *Edmontosaurus* sample, although a small carbonyl absorption band is just visible (Figure 3 inset). The intensity ratio for carbonyl over phosphate (indicated by “CO/P”) is used as a proxy for collagen abundance^38,39^ but does not guarantee that the carbonyl moiety is from collagen. For the turkey sample CO/P was 0.455 and 0.065 for UOL GE0.1.

### XPol

Cross-polarized light, or “crossed-polar” microscopy has been used to image stained skin collagen for quantification of collagen density and unstained bone collagen.^40^ The architecture of bone lamellae can be observed under polarized light microscopy since bone is optically anisotropic (birefringent). It is the composite of collagen fibers and bioapatite crystallites in regular patterns that gives bone its birefringence.

**Figure 4:**
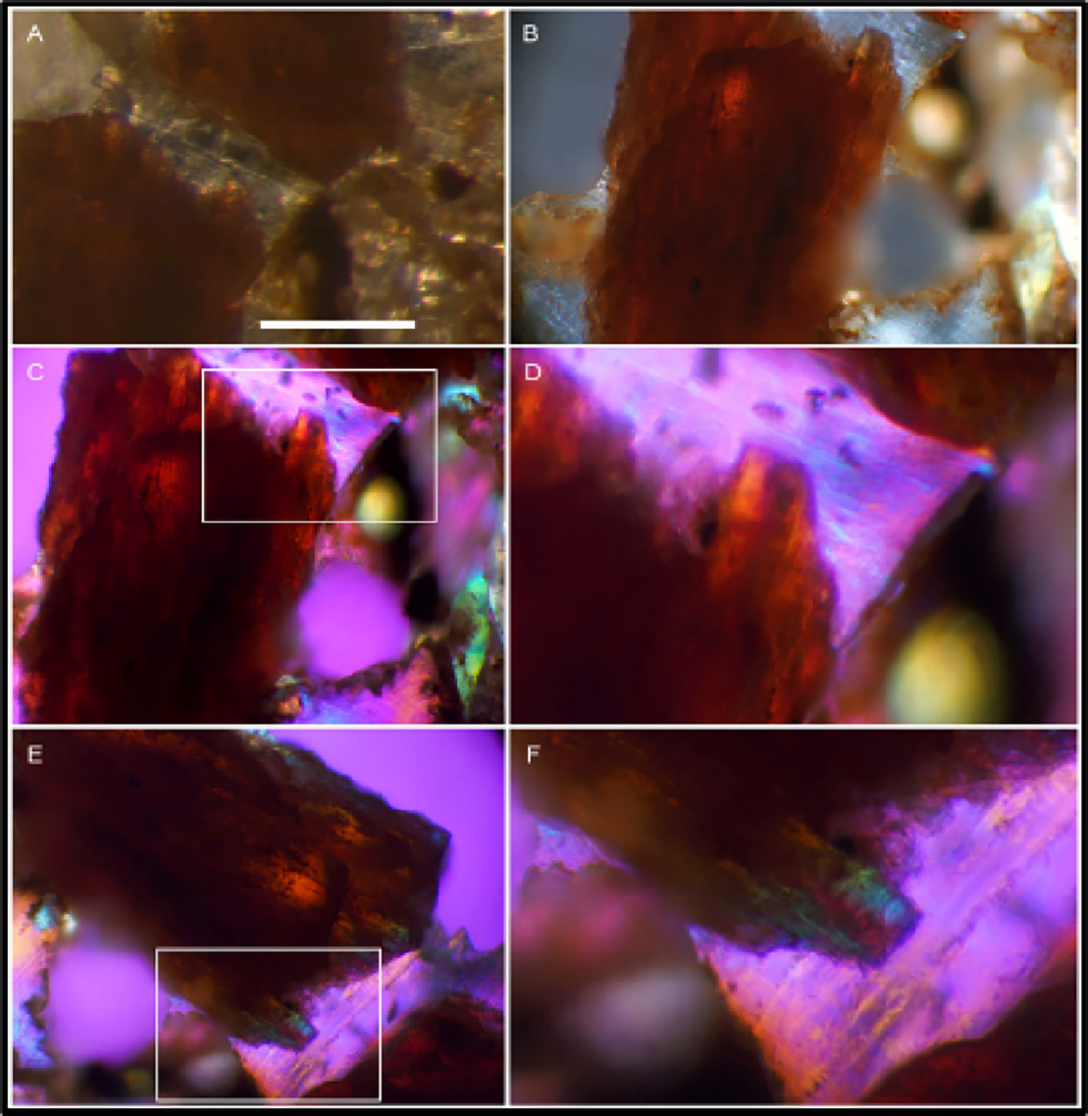
Cross-polarized light (XPol) micrographs of *Edmontosaurus* UOL GEO.1 thin sections. (A) Stereomicrograph includes the region of interest (ROI) seen as a brown peninsula-shaped edge of a bone fragment left of center. Scale bar 200 μm, image collected at 100X. (B) Bone fragment under crossed polars oriented to extinction. Thickness of opaque regions (at approximately 0.15 mm) of tan-colored bone occlude light, but the thinner margins permit more light and appear gold. (C) Same bone fragment in XPol with a first order red filter. Image 200X. (D) Expanded view of inset shows the ROI with gold hue. (E), (F) Same bone fragment after stage was rotated clockwise approximately 100 degrees reveals the gold areas turned blue, green, and lavender. This birefringence is consistent with intact bone collagen remnants.

XPol images of UOL GEO.1 bone tissue (Figure 4) revealed differing characteristics of color within two distinct microscopic regions. Hard-edged, angular green shapes are interpreted as calcite inclusions within osteonic lumens. However, a minority of regions that were once fresh bone tissue also show birefringence. Unlike the calcite inclusions that can occur in lumens, these regions occur within the bone matrix. They contain small, dark lacunae that once held osteons.

Birefringence within formerly fresh bone appears reddish-gold under crossed polars (Figure 4B). With a first order red filter, XPol revealed gold-colored regions (Figure 4C inset and Figure 4D) that turned blue-green when rotated over 90°. This birefringence characterizes the collagen-bioapatite crystallinity that pervades fresh bone. This appears to occur only in patches within the fossil.

Two options present themselves to help interpret the observed birefringence. In one, collagen has decayed from all of the *Edmontosaurus* bone matrix. Since bioapatite crystallites rapidly disperse upon collagen degradation,^10^ some other cementing agent would have replaced the role of collagen in holding those crystallites in their original positions and pattern. This scenario would require the exogenous cementing agent, to replace the collagen only within the still-birefringent regions. These minerals would have permineralized pore spaces such as the osteonic lumens before penetrating only a minority of bone matrix.

Three deficiencies with this diagenetic scenario emerge. First, the requirement of a cementing agent to move in place of collagen while maintaining the spatial positioning of bioapatite crystallites is unlikely, with randomization or indeed loss of crystallites a more likely outcome. Second, the water required to transport dissolved ions that precipitate into minerals would have facilitated degradative chemistry alongside physical dispersion and transport of original bone collagen and/or bioapatite. Lastly and equally unlikely, the replacement cementing mineral would exactly replicate original crystallite positioning so as to retain bone microstructures including lamellae (seen in other samples) and lacunae as seen in Figure 4.

A second option involves retention of sufficient original collagenous remnants to preserve crystallites in life position. The many published descriptions of biomolecular remnants in fossils strongly suggest that original protein may persist in Cretaceous bone. Indeed, at least ten reports describe remnant osteocytes liberated by dissolution from fossil bone,^1,41−49^ showing some preservation of original organics.

A more parsimonious explanation for the regions within the extracellular matrix (ECM) that retain a degree of birefringence is that they retain remnants of original collagen sufficient to hold some crystallites in their original patterns. Accordingly, the regions within bone that have no birefringence would represent zones where collagen has completely decayed and thus where crystallites have dispersed.

**Figure 5:**
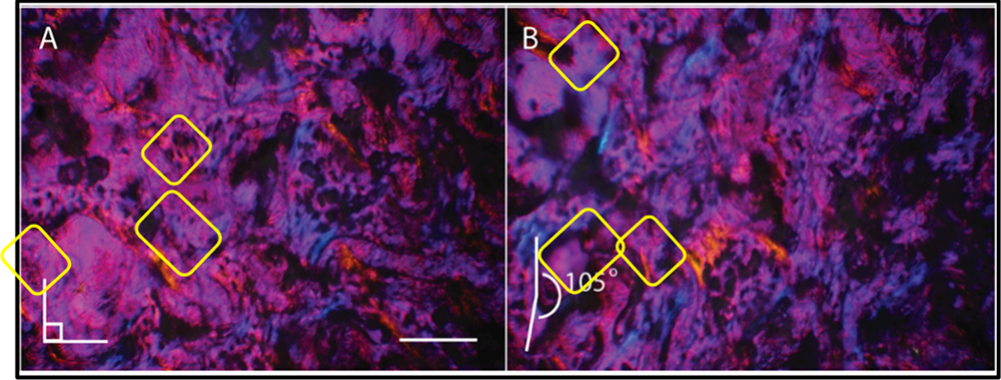
XPol with first order red filter micrograph of artificially decayed *Meleagris* proximal epiphysis of femur. (A) Gold and blue regions suggest bone collagen remnants. Scale bar 200 μm. (B) Gold regions turn blue and blue regions turn gold after rotating the stage showing birefringence that confirms collagen remnants. Lavender regions with little to no birefringence we interpret as collagen-depleted, similar to the patchy pattern of collagen that XPol reveals in fossil bone.

Turkey bone was artificially decayed at high temperature. Similar birefringence to fossil bone was observed under XPol with a first order red filter (Figure 5). No permineralization was observed in osteons, as expected, since our bone decay procedure did not include dissolved ions. In this case, almost-black areas remain dark after rotating the sample at an approximate right angle (105°), making them no longer birefringent. Bioapatite would have dispersed from these areas as high temperatures accelerated the collagen decay. However, microregions traded gold for blue and *vice versa* upon rotation, in a similar way to the fossil. These results are also consistent with the retention of collagen remnants in birefringent microregions in both artificially decayed turkey and actually decayed *Edmontosaurus* bone.

### LC-MS/MS Bottom-Up Proteomics

**Figure 6:**
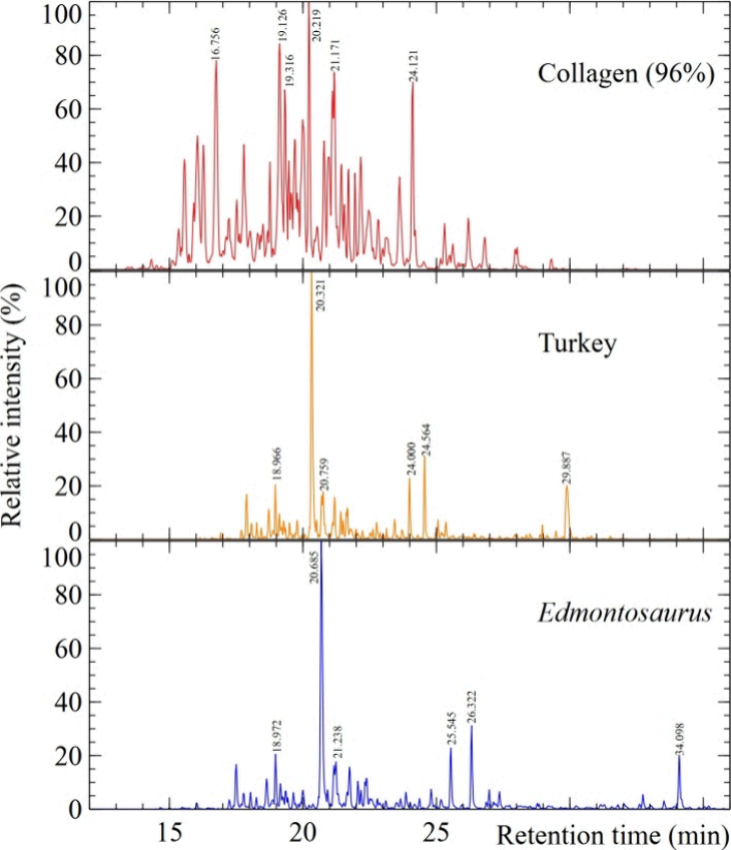
BPI chromatograms of *Edmontosaurus* (blue line, bottom) and *Meleagris* (turkey, orange line, middle) bone overlaid with that obtained from bovine collagen 96% (red line, top). Retention times for the highest six peak intensities are individually labeled.

15 min survey analyses were used to determine the amount of each sample required to obtain comparable signal intensities. The base peak intensity (BPI) for 30 min chromatograms of the *Edmontosaurus* and turkey bone samples are shown in Figure 6 along with authentic bovine collagen spectra.

The samples were digested with trypsin. The resulting fragments were ionised with a nanospray ionization source (nESI). Ions with charge between +2 and +5 were filtered to enter the mass spectrometer, which they do at different times depending on their retention affinity with the chromatography column, before being mass analyzed by the detector (Figure 6). The resulting ion masses are then compared with those in existing databases as described in the database searching methods section. There is an overall similarity between the chromatograms for the turkey and the *Edmontosaurus* samples and a close match between the retention times for the highest peaks (at 20.321 for turkey and 20.685 for*Edmontosaurus*) and those peaks immediately preceding and following. The difference in retention times is possibly due to differing bone matrices having different binding affinities to the column.^50^

Analysis of the data sets revealed six collagen-derived peptides in the *Edmontosaurus* sample, with errors ranging from 1.3–3.6 ppm (Table 1) and oxidized at a minimum of one position (always on proline, i.e., hydroxyproline). The ppm error is calculated by the difference between the measured mass and theoretical (database) mass divided by the theoretical mass. All these sequences correspond to peptides reported in the SwissProt database for *Brachylophosaurus canadensis*, another duck-billed dinosaur that together with *Edmontosaurus* is classed in the Hadrosauridae family. Five of the sequences are from the collagen alpha-1(I) chain (length: 113 amino acids, mass: 9664 Da), in total accounting for covering 73.45% of the entire sequence (positions 19–33 and 64–78 absent). The remaining detected peptide (*m*/*z* 805.38074) accounts for 50% coverage of the collagen alpha-2(I) chain (length: 36 amino acids, mass: 3122 Da), with positions 19–36 unaccounted. Three sequences (rows 1, 4, and 5 of Table 1) are also reported for a *T. rex* sample from the Hell Creek formation.^51^ Most of the PTMs on these *Edmontosaurus* sequences are oxidation, but we also note deamidation of N (Asparagine) and Q (Glutamine) amino acids: in 85/150 peptides and 55/95 peptides, respectively.^52,53^ Deamidation for our turkey sample is significantly lower (32/440 peptides).

**Table 1:** *Edmontosaurus* (UOL GEO.1) Peptides Detected from Alpha-1 and Alpha-2 Type I Collagen.^a^

Collagen ions (u)	Data search match (u)	ppm error	Protein	Assigned sequence	Residue assignment
786.8897	786.8918	–2.7	P86289|CO1A1_BRACN	GATGAPGIAGAPGFPGAR	1–18
766.87427	766.8762	–2.5	P86289|CO1A1_BRACN	GETGPAGPAGPPGPAGAR	96–113
795.90826	795.9097	–1.8	P86289|CO1A1_BRACN	GLTGPIGPPGPAGAPGDK	46–63
730.34717	730.3498	–3.6	P86289|CO1A1_BRACN	GSAGPPGATGFPGAAGR	79–95
581.80048	581.8018	–2.3	P86289|CO1A1_BRACN	GVQGPPGPQGPR	34–45
805.38074	805.3818	–1.3	P86290|CO1A2_BRACN	GSNGEPGSAGPPGPAGLR	1–18

a Masses were calculated using ExPASy PeptideMass and configured as follows: cysteine residues treated with iodoacetamide, methionine residues oxidized, monoisotopic mass, allowance for a maximum of one missed tryptic cleavage, mass between 500–unlimited Dalton (u), z equal to 1+ or 2+ selected.

Supplementary Table S3 lists a total of at least 41 discovered collagen sequences from UOL GEO.1. It also lists PTMs for the first eight accessions and refers to the raw data (login details are on page 1 of the Supporting Information. See also Figure S4 caption). These matched a mixture of extant and extinct fauna found in the SwissProt database. The most abundant of these matched peptides were from domestic chicken (*Gallus gallus*), followed by American mastodon (*Mammut americanum*). In addition, peptides from other proteins were detected in the *Edmontosaurus* sample. Over a hundred actin peptides were found. Furthermore, 61 hemoglobin, 158 histone, and 92 tubulin peptides were also detected.

The Basic Local Alignment Search Tool (BLAST) analysis (Supplementary Table S7) revealed that the collagen sequence does have genera-specific differences based on comparison with extant taxa and recurring sequence similarities based on functional constraints. There is also the question of variabilities caused through diagenesis, which may blur genera-specific residues. The similarity of the UOL GEO.1 sequence to both extant and extinct taxa are not therefore unexpected.

Several sequences, including those from chicken (*Gallus gallus*) and dog (*Canis lupus familiaris*), returned sequence identity scores at least as high as hadrosaur (*Brachylophosaurus canadensis*). The question arises as to whether such results indicate contamination. Modern humans of course associate with chicken and dog, but not likely with rat (*Rattus norvegicus*) and not at all with mastodon (*Mammut americanum*), both of which also scored higher than *B. canadensis*. Since it is not possible for mastodon sequence to have entered the analysis stream via modern contamination, then alternate explanations for it and accompanying taxa should be sought. If previously tested samples had included our reported taxa, such a finding would provide evidence for contamination through holdover within instrumentation. However, fruit fly (*Drosophila melanogaster*) samples preceded our dinosaur, samples and no *Drosophila* peptide sequence matches occurred in our data set. However, different database algorithms can assign the same peptide sequences to different taxa. Furthermore, peptide sequences can be remarkably similar among widely different taxa,^54,55^ even up to the phylum level.^56^ Thus, it is not unreasonable to expect and find almost identical sequences among differing taxa.

The turkey bone data checked against the UniChick database (Supplementary Table S4) gave 55.33% coverage of collagen alpha-1(I) chain and 74.69% coverage of the alpha-2(I) chain. The UniTurkey database contains no matches for type I collagen (Supplementary Table S5). No *Brachylophosaurus* peptides were found in the turkey bone data set (compared against UniChick and UniTurkey databases). A majority (185) of the 188 peptides found in bovine tendon (96% pure) collagen matched that of *Bos taurus*, accounting for 98.40% of the amino acid sequence (Supplementary Table S6).

### LC-MS/MS of Hydroxyproline

Under ESI conditions, Hyp_be_ (butyl ester of hydroxyproline) produces an intense parent ion at *m*/*z* 188 (MH^+^), which upon fragmentation under the specified MS/MS conditions, yields fragment ions at *m*/*z* 132 and 86. The former likely results from loss of the butyl side chain (C_4_H_8_) with a proton transfer to the CO group: thus, (C_5_H_9_NO_3_^+^H)^+^ would be the corresponding elemental composition. Further neutral loss of 46 u (H_2_CO_2_) through proton transfer and cleavage of the acid group would create the *m*/*z* 86 fragment (C_4_H_8_NO^+^).

**Figure 7:**
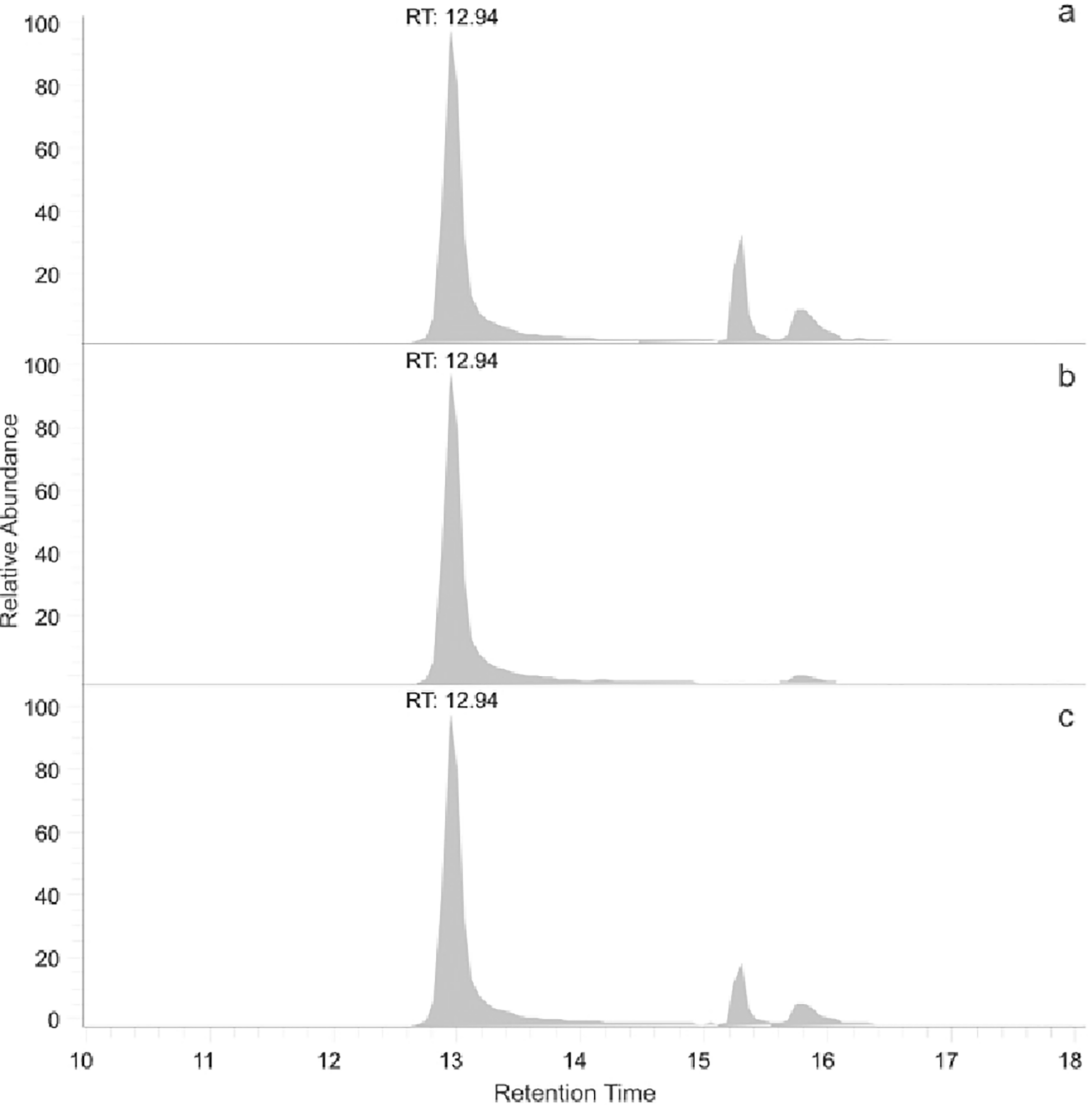
Chromatogram for Hyp from one of the *Edmontosaurus* bone samples. Upper trace (a) shows the chromatogram for the transition 188–132, the middle trace (b) for 188–86, and the bottom trace (c) is the sum of both transitions.

Following injection of the derivatized bone samples, reconstructed ion traces for *m*/*z* 188–132 and 188–86 transitions consistently showed significant peaks at or near the retention of authentic Hyp_be_ (Figure 7, top two traces). Despite all precautions taken, the negative controls always revealed a peak for Hyp. However, the intensity of this peak was significantly less than what was obtained for the bone samples (Table 2).

**Table 2:** Average Concentration of Hydroxyproline in Five Independent Experiments (nmol/sample)

	**Negative control**	*Edmontosaurus***bone**	**Turkey bone**	**Bovine collagen**
	0.12	2.71	537.90	151.81
	1.41	9.25	197.07	66.38
	1.18	6.18	145.52	87.89
	0.08	0.03	434.03	519.14
	0.95	1.99	232.28	87.89
Mean	0.55	2.94	309.36	182.62
Stdev	0.56	3.53	168.20	190.83
N	5	5	5	5

A cochromatography experiment was performed because the Hyp analyses were marked by a greater than usual variation in retention times. After analyzing the individual samples, the dinosaur bone and authentic Hyp_be_ samples were mixed, and the result from the mixed sample revealed a single sharp peak. This cochromatography experiment showed the material in dinosaur bone was chromatographically indistinguishable from Hyp_be_.

Furthermore, the ratios of the peak areas for the two transitions (188–132, 188–86) from authentic Hyp_be_ and the peak from derivatized bone extract were the same (Table 3).

**Table 3:** Ratio of the Peak Areas for the Two Transitions (188–132/188–86) in Edmontosaurus Fossil Bone

	**Authentic Hyp**_**be**_ (*n* = 10)	**Assigned Hyp**_**be**_**peak from bone samples** (*n* = 6)
Mean	1.20	1.21
Stdev	0.02	0.06

Further testing of the validity of the assertion that the chromatographic peak assigned as Hyp_be_ in the fossilized samples came from accurate mass measurements completed with the orbitrap as the chromatographic detector. The measured mass of the parent ion from the fossilized bone sample was recorded at *m*/*z* 188.12802, that is within 0.5 ppm of the theoretical calculated value (calculated for C_9_H_1_7NO_3_^+^H^+^, 188.12812), and this lies within the instrument tolerance.

Finally, the Hyp_be_ concentration in the fossilized *Edmontosaurus* bone sample was estimated by interpolating peak areas for the 188–132 transition using a standard curve constructed from a series of samples of decreasing Hyp_be_ concentration. The peak areas in the standard reference curves covered the range of peak areas in Negative controls and the *Edmontosaurus* bone samples, so the quantitation in these cases was achieved by interpolation from the standard curve. The Hyp peak areas in the Turkey bone and Bovine collagen samples were outside the range of the standard curves, so in these cases quantitation was achieved by extrapolation.

Hyp_be_ was reliably detected and quantified in six separate 1 mg samples taken from the same bone specimen, with concentrations ranging from 6.7 to 41.7 nmoles of Hyp_be_/gram of bone (Table 4), a result reflecting an uneven distribution of collagen within the fossilized sample consistent with XPol imaging which also revealed an uneven distribution of bone collagen within microscopic fossil bone regions.

**Table 4:** Hydroxyproline (Hyp_be_) Concentration in *Edmontosaurus* Fossil Bone Calculated Based on Transition 188–132

**Sample ID**	**Nanomoles Hyp/gram of bone**
1	41.7
2	19.1
3	6.7
4	8.9
5	15.4
6	8.6

## Discussion

The discovery of soft tissue in dinosaur remains has been controversial due to conflicting explanations of contamination versus endogeneity for the observed results.^28,57,58^ In an attempt to look for contamination, our novel approach attempts to quantify hydroxyproline. The hypothesis being that if prior techniques used to characterize fossil bone collagen were subject to false positives via contamination, e.g., recent noncollagen, collagen look-alikes, or by residual collagen trapped within instrumentation,^59,60^ then it would be less likely to detect and quantify Hyp. However, in multiple runs taken from separate sample extracts, it was possible to quantify Hyp. This result is more consistent with the hypothesis of preserved collagenous remnants and at the same time makes claims of contamination more difficult to defend.

Another novelty of our methodology is that by quantifying, as well as sequencing, a sense may be gained for how decayed the collagen is. If the sequenced collagen is contamination by recent sources, then the sequence would be largely complete (not having been around long enough for partial or severe chemical decay) and the yields would be relatively closer to that of fresh bone. Instead, we find short sequence fragments and lower Hyp yields, both independently consistent with ancient and decayed, not modern, collagen.

To date, collagen sequence data has been published from limb bones, i.e., *T. rex* and *B. canadensis* femurs. Here for the first time, we show independent, partial matches to those sequences but extracted from a sacrum. A further novelty is that, to date, no researchers have used protein sequencing in combination with XPol. Until now, XPol has been used to visualize collagen in fresh bone, not ancient bone. This is possibly due to the expectation that mineralization during diagenesis would have replaced the birefringent property of ancient collagenated bone. For the first time XPol results showing collagen-like birefringence in combination with collagen sequence for dinosaur establishing XPol as another tool to characterize fossil bone collagen.

Previous studies using FTIR of Jurassic^24^ and Cretaceous^23^ dinosaurs have shown the organic amide I group around the absorption band 1650 cm^–1^ and the phosphate vibrations representing apatite are found between 960–1100 cm^–1^.^32,36,39,61^ Here such results are only confirmed in modern turkey bone. In completely mineralized bone, the CO maximum would be indistinguishable from the baseline absorbance. In the *Edmontosaurus*, the CO absorption maximum is above baseline with a CO/P ratio of 0.065 cm^–1^, consistent with residual organic material, but not collagen conclusively.

Microscopic regions within UOL GEO.1 retain birefringence characteristic of the original collagen/bioapatite bone constituents. They also resemble similar regions within artificially decayed *Meleagris* bone. Areas that appear dark and purple are no-longer birefringent and surround the birefringent regions. If high temperatures were extended in the *Meleagris* experiment, birefringence would eventually give way to nonbirefringent regions, consistent with biochemical decay of bone collagen and its subsequent release and randomization of bioapatite crystallites. Therefore, it is possible that the nonbirefringence seen in much of the field-of-view corresponds to decayed bone collagen in both the artificially and actually decayed (fossil) bone samples.

The similarity between birefringent patterns for *Meleagris* and *Edmontosaurus* bone are consistent with the concept that endogenous collagenous remnants with sufficient integrity to hold enough bioapatite crystallites in their original, regular arrangements continue to cause birefringence in both these samples of bone tissue. However, since XPol cannot directly report molecular data, any conclusions regarding molecular preservation require independent analyses to confirm collagen remnants. If confirmed independently in the same fossil bone sample by e.g. MS analysis, then XPol offers the possibility of both describing and spatially mapping microscopic collagen regions and decay patterns in ancient bone.

Hydroxyproline (Hyp) is found—but uncommon—in few proteins other than collagen,^62^ but comprises somewhere between 4–10% of collagen residues in extant vertebrates. The presence of hydroxyproline in the fossils is consistent with a collagenous origin.^63^ Because collagen is by far the most abundant protein in bone tissue, Hyp is targeted as an indicator of collagen and our results verify the presence of Hyp in acid-digested samples. This otherwise unusual amino acid constitutes 9.6, 7.8, and 4.0 residue percent of collagen from rat, bovine, and codfish, respectively.^64^ In our experiments for the first time, the amount of collagen in Mesozoic dinosaur bone is quantified by singling out authentic Hyp, in samples of known provenance.

MS is the preferred method for protein identification providing unparalleled sensitivity and specificity.^65,66^ MS indicated the presence of organic materials (alpha chains) in *T. rex*.^20^ At that time these were not referenceable against a database with dinosaurian peptides. Later, *Brachylophosaurus* data also yielded peptide chains and the presence of Hyp.^21,22^ This paper is the first wholly independent confirmation of these previous conclusions via similar results in *Edmontosaurus* UOL GEO.1, howbeit on a relatively limited data set. Our bottom-up proteomic analyses revealed a total of at least 41 collagen polypeptides (Supplementary Table S3). The focus of the results in this paper is on the peptides assigned to *Brachylophosaurus* alpha-1 and alpha-2 helices. It is probable that similar taxa (*Brachylophosaurus* and *Edmontosaurus*) had many proteins in common. For instance, two collagen alpha-1 (I) chain peptides (residue assignments 1–18 and 79–95) found in the *Edmontosaurus* were also discovered in the *Brachylophosaurus* both with modifications on the same prolines.^22^ In total, five revealed polypeptides are assigned to *Brachylophosaurus canadensis* collagen alpha-1 (I) helix; a sixth one belongs to the collagen alpha-2 (I) helix of the same. These peptides, some unique to dinosaur, can therefore be regarded as confirmation of original endogenous collagen rather than contamination from any extant creature. The scarcity of post translational modifications (PTMs) is evidence of exceptional preservation for UOL GEO.1.

## Summary

Detection of soft tissues (e.g., proteins) in fossil bones is a growing field of study and this paper contributes to the list of such findings. Corroborating results from a novel combination of three independent analytical techniques are presented which taken together provide experimental evidence for the conclusion that collagenous protein remnants in some dinosaur bones are original (endogenous) to the fossils and thus providing further evidence addressing this long-standing controversy in the scientific literature.

## Data Availability

The mass spectrometry proteomics data generated and/or analyzed during the current study have been deposited to the ProteomeXchange Consortium via the PRIDE^67^ partner repository with the data set identifier PXD048810 (login details on page 1 in Supporting Information).

## Supporting Information Available

Links to 3D models, additional experimental details, chromatograms and protein match summaries, annotated peptides, BLAST of *Edmontosaurus* peptide sequences (PDF)
