# Correction to “Evidence for Endogenous Collagen in *Edmontosaurus* Fossil Bone”

**DOI:** 10.1021/acs.analchem.5c02452

**Published:** 2025-04-30

**Authors:** Lucien Tuinstra, Brian Thomas, Steven Robinson, Krzysztof Pawlak, Gazmend Elezi, Kym Francis Faull, Stephen Taylor

The spectra provided for Turkey in the original article was mistakenly the *Edmontosaurus* data set reformulated and stretched by the Origins graph plotting program. This was the result of a transcription error from the raw data file from the Thermo Q Exactive Orbitrap into the Origins program used to generate Figure 6.

**Figure 6:**
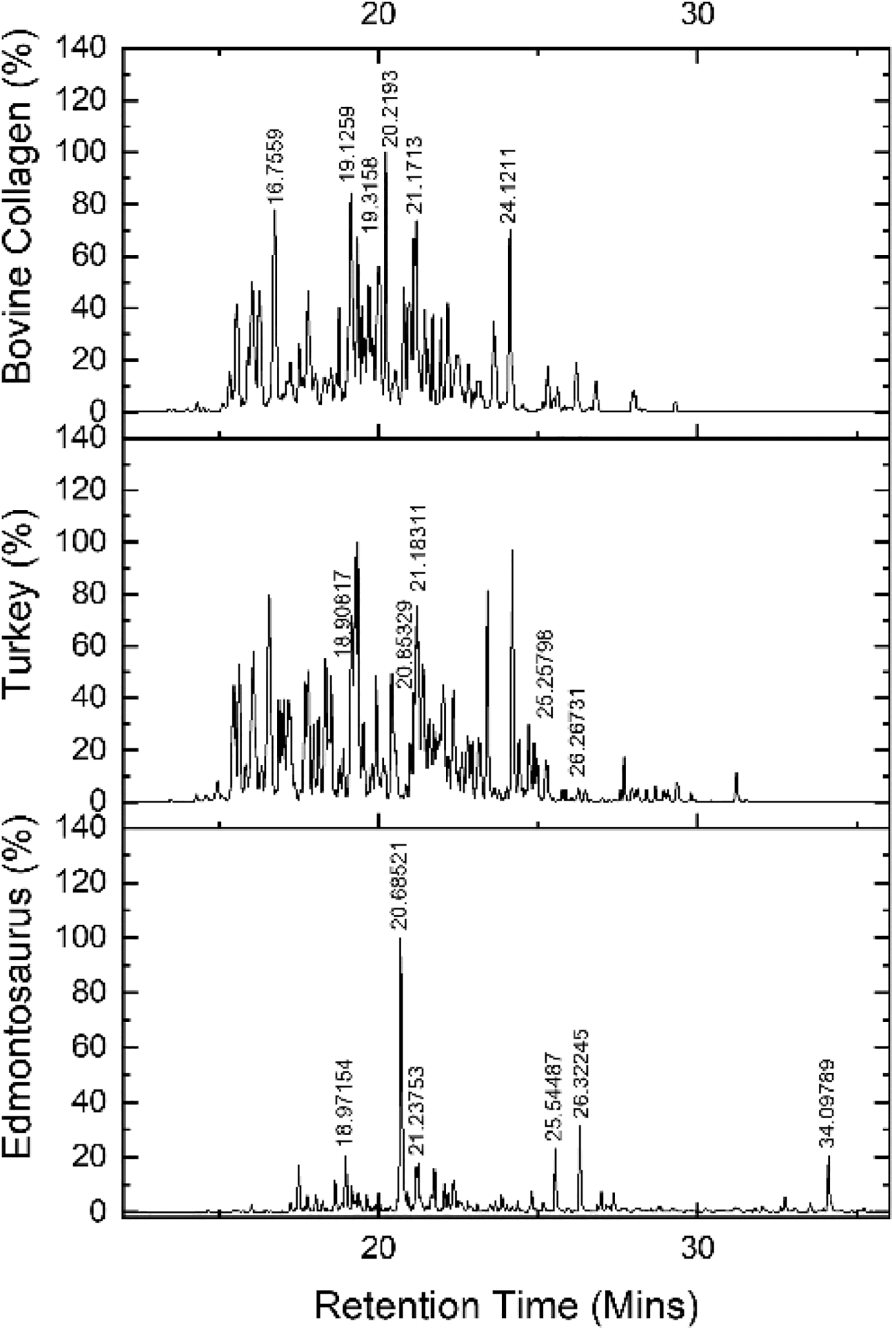
BPI chromatograms of Edmontosaurus (bottom) and Meleagris (turkey, middle) bone overlaid with that obtained from bovine collagen 96% (top). Retention times for the highest peak intensities are individually labeled.

A corrected copy of Figure 6 is shown above. The raw data are as presented in the Supporting Information of our paper and are linked to below.

The conclusions of the paper we believe are unaffected from this correction. In our view, the new figure provides stronger evidential support for the conclusions drawn regarding endogeneity of dinosaur collagen.

Fifteen min survey analyses were used to determine the amount of each sample required to obtain comparable signal intensities. The base peak intensity (BPI) for 30 min chromatograms of the *Edmontosaurus* and turkey bone samples are shown in Figure 6 along with authentic bovine collagen.

There is an overall similarity between the chromatograms for the turkey and the bovine collagen samples and matching retention times for some of their highest peaks. The *Edmontosaurus* sample has fewer peaks than the other two analytes as might be expected from a degraded sample. Five of the six highest *Edmontosaurus* peaks, at 18.97, 20.69, 21.24, 25.54, and 26.32 min, match peaks in the turkey bone, although with some differences in retention times (between −0.17 and +0.29 min). This result is consistent with the presence of collagen in all samples.

The raw data for the Figure 6 may be found on the PRIDE partner repository and that for the LC–MS of the hydroxyproline (Figure 7) may be found on the data catalogue at the University of Liverpool: 10.17638/datacat.liverpool.ac.uk/3009.

