# Supplementary material for: Evidence for Endogenous Collagen in Edmontosaurus Fossil Bone
Source: Anal Chem. 2025 Jan 17;97(5):2618–28. doi: 10.1021/acs.analchem.4c03115 (PMC11822843; doi:10.1021/acs.analchem.4c03115)
Supplement: Supplementary file 1 — ac4c03115_si_001.pdf [file ac4c03115_si_001.pdf]

Supporting Information

Evidence for endogenous collagen in *Edmontosaurus* fossil bone

Lucien Tuinstra[1], Brian Thomas[1], Steven Robinson[2], Krzysztof Pawlak[2], Gazmend Elezi[3], Kym Francis Faull[4], & Stephen Taylor[1]*

[1] Department of Electrical Engineering and Electronics, University of Liverpool, L69 3BX, UK
[2] Materials Innovation Factory, University of Liverpool, L7 3NY, UK
[3] Pasarow Mass Spectrometry Laboratory, Jane and Terry Semel Institute for Neuroscience and Human Behaviour and Department of Psychiatry & Biobehavioral Sciences, David Geffen School of Medicine, University of California, Los Angeles, USA
[4] Former Director of Pasarow Mass Spectrometry Laboratory, Jane and Terry Semel Institute for Neuroscience and Human Behaviour and Department of Psychiatry & Biobehavioral Sciences, David Geffen School of Medicine, University of California, Los Angeles, USA
* Prof Stephen Taylor, 🖂 S.Taylor@liverpool.ac.uk

Contents

Table S1: Links to 3D model of the *Edmontosaurus sp*. sacrum………………………….. S-2
Table S2: Details of the various ranging runs ……………………………………………….. S-2
Figure S1 BPI (3.18e9) chromatogram for *Edmontosaurus sp.* (UOL GEO.1)…………… S-2
Table S3: Protein match summary for *Edmontosaurus* (UOL GEO.1) (SwissProt)……… S2-S6
Figure S2 BPI (2.80e9) chromatogram for modern turkey *(Meleagris gallopavo*)……….. S-7
Table S4: Protein match summary for modern turkey (*M. gallopavo*) (UniChick)……….. S7-S8
Table S5: Protein match summary for modern turkey (*M. gallopavo*) (UniTurkey)………. S8
Figure S3 BPI (2.62e9) chromatogram for bovine (*Bos taurus*) tendon collagen (96%)… S9
Table S6: Protein match summary for bovine (*B. taurus*) tendon collagen (96%)……….. S9
Figure S4 Annotated peptides corresponding to Table 1 in the manuscript (and appear in the same order from top to bottom)……………………………………………….…. S10-S11
Table S7: BLAST of peptide sequences discovered in *Edmontosaurus* (UOLGEO.1)……. S12

Data availability statement

The mass spectrometry proteomics data generated and/or analysed during the current study have been deposited to the ProteomeXchange Consortium via the PRIDE [61] partner repository with the dataset identifier PXD048810.

Login details: https://www.ebi.ac.uk/pride/login

Username: reviewer_pxd048810@ebi.ac.uk

Password: rRwI5SAd

Sample

The *Edmontosaurus sp.* fossil bone was found in Harding County, SD, USA and acquired by a licensed collector (Alan Stout). The photogrammetry model was reconstructed using 1061 images in Agisoft Metashape software version 1.7.2. Sony alpha ILCE-6400 cameras were used with a Sony 50 mm Macro F2.8 FE Lens. Even, diffuse lighting was used and images were saved in both RAW and fine JPG format, with JPGs used for the reconstruction of the models. Due to the level of granular detail in the objects, and the controlled conditions, high resolution (100% of image resolution) was used for the camera alignment process. The final models varied between 2–5 million faces. The models were down-sampled to 64 thousand faces for online loading speeds while retaining adequate accuracy for general inspection.

Table S1: Links to 3D model of the *Edmontosaurus sp. sacrum*

https://sketchfab.com/3d-models/large-fused-frag-scaled-1b3ec27799434e2cb82d04d0a98b720f
https://sketchfab.com/3d-models/long-frag-scaled-7072c0618f1e4e428fb058e5196b6b47
https://sketchfab.com/3d-models/small-frag-scaled-545a6dc5ef974aa295caf680c35f3542

Table S2: Details of the ranging runs performed to ascertain the appropriate volume of each sample to load onto the instrument.

Sample	Ranging Runs Performed (sample dilutions to check signal intensity)	Method Used	Injection Volume
Edmontosaurus	1:1000, 1:10	‘Blank 30-min’	1 µL
Modern Turkey	1:1000	‘Blank 30-min’	1 µL
Bovine Collagen	1:1000	‘Blank 30-min’	1 µL

Results

Sample 1: *Edmontosaurus sp.* (UOL GEO.1)

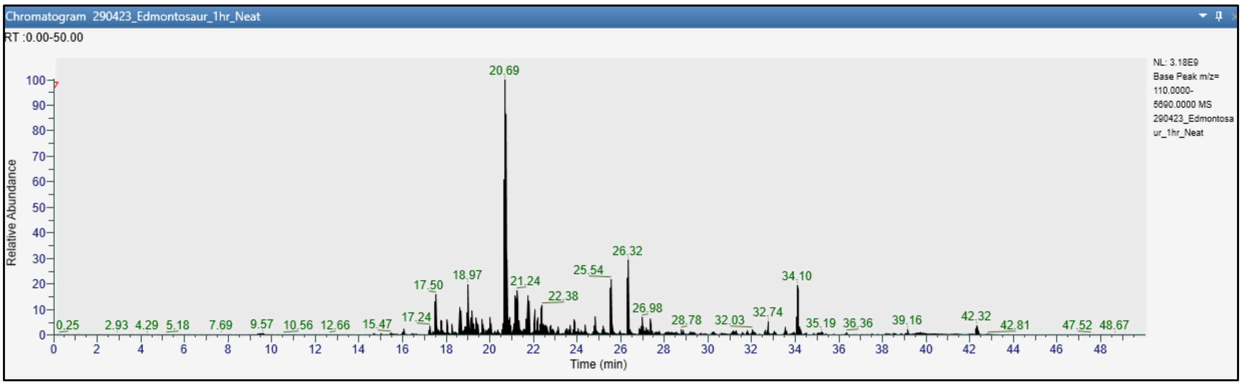

Figure S1. BPI (3.18e9) chromatogram for *Edmontosaurus sp.* (UOL GEO.1)

Table S3: Protein match summary for *Edmontosaurus* (UOL GEO.1). Database Searched: SwissProt (Entries: 564,638)

Accession	Score	Sequence Coverage (%)	No. of Pep-tides	No. of unique Seq’s	Description	Assigned Sequence
**P02467|CO1A2_CHICK**	361.9	32.43	27	24	Collagen alpha-2(I) chain OS=Gallus gallus	GNVGLAGPR<br>VGPIGPAGNR<br>EGPVGFP(+15.99)GADGR<br>NGLP(+15.99)GPIGPAGVR<br>GEGGPAGPAGPAGAR<br>GLP(+15.99)GQP(+15.99)GSP(+15.99)GPAGK<br>GAP(+15.99)GLRGDTGATGR<br>GIP(+15.99)GPP(+15.99)GPAGPSGAR<br>GLHGEFGVP(+15.99)GPAGPR

						GLP(+15.99)GAIGAP(+15.99)GPAGGAGDR<br>TGEQGIAGPP(+15.99)GFAGEK<br>GDAGPP(+15.99)GM(+15.99)TGFP(+15.99)GAAGR<br>GDP(+15.99)GPVGA(sub P)VGPAGAFGPR<br>GEIGPAGNVGPTGPAGPR<br>GLP(+15.99)GESGAVGPV(sub A)GPIGSR<br>GPSGPVGSP(+15.99)GPNGAP(+15.99)GEAGR<br>GEP(+15.99)GAAGPP(+15.99)GPP(+15.99)GPSGEEG<br>K<br>VGP(+15.99)P(+15.99)GPAGITGPP(+15.99)GPP(+15.9<br>9)GPAGK<br>GEP(+15.99)GNVGPAGAP(+15.99)GPAGPGGIP(+15.9<br>9)GER<br>GLP(+15.99)GIAGATGEP(+15.99)GPLGVSGPP(+15.99<br>)GAR<br>GAPGP(+15.99)EGNNGAQGPP(+15.99)GVTGNQGAK<br>GETGPTGAIGPIGASGPP(+15.99)GPVGAAGPAGPR<br>GLP(+15.99)GAIGAP(+15.99)GPAGGAGDRGEGGPAG<br>PAGPAGAR<br>GLAGPQGPR
**P02457|CO1A1_CHICK**	355.3	25.74	26	12	Collagen alpha-1(I) chain OS=Gallus gallus	GRP(+15.99)GPSGPAGAR<br>GAP(+15.99)GPPGAVGAAGK<br>GAP(+15.99)GPP(+15.99)GAVGAAGK<br>GEAGPP(+15.99)GPAGPTGAR<br>GEP(+15.99)GPAGLP(+15.99)GPAGER<br>GAAGLPGAKGDR(+15.99)GDP(+15.99)GPK<br>DGEAGAQGPPGPTGPAGER<br>DGEAGAQGPP(+15.99)GPTGPAGER<br>NGDRGETGPAGPAGPP(+15.99)GPAGAR<br>GSP(+15.99)GADGPIGAP(+15.99)GTP(+15.99)GPQGI<br>AGQR<br>GFSGLQGPP(+15.99)GPP(+15.99)GAP(+15.99)GEQG<br>PSGASGPAGPR<br>GLTGPIGPP(+15.99)GPAGAP(+15.99)GDKGEAGPP(+<br>15.99)GPAGPTGAR
**P0C2W8|CO1A1_MAMAE**	306.7	21	15	2	Collagen alpha-1(I) chain OS=Mammut americanum	GPAGPQGPSGAP(+15.99)GPK<br>GETGPAGPAGPAGPAGVR
**P85154|CO1A2_MAMAE**	281.5	17.12	11	5	Collagen alpha-2(I) chain OS=Mammut americanum	GEAGPAGSAGPAGPR<br>GPP(+15.99)GQSGAAGPTGPIGSR<br>GPSGDSGRP(+15.99)GEP(+15.99)GVM(+15.99)GPR<br>GIN(sub P)GPVGAAGATGAR<br>GEP(+15.99)GPAGSVGPVGAVGPR
**P02454|CO1A1_RAT**	278.2	10.05	11	2	Collagen alpha-1(I) chain OS=Rattus norvegicus	GEP(+15.99)GPAGVQGPP(+15.99)GPAGEEGK<br>GEPGP(+15.99)AGVQGPP(+15.99)GPAGEEGK<br>GEP(+15.99)GPAGVQGPP(+15.99)GPAGEEGKR
**O46392|CO1A2_CANLF**	244.7	8.05	7	1	Collagen alpha-2(I) chain OS=Canis lupus familiaris	GLP(+15.99)GEFGLP(+15.99)GPAGPR
**P86289|CO1A1_BRACN**	230.9	86.73	6	1	Collagen alpha-1(I) chain (Fragment) OS=Brachylophosaurus canadensis	N(sub G)ETGPAGPAGPP(+15.99)GPAGAR<br>GVQGPP(+15.99)GPQGPR<br>GETGPAGPAGPP(+15.99)GPAGAR<br>GETGPAGP(+15.99)AGPP(+15.99)GPAGAR<br>GATGAP(+15.99)GIAGAP(+15.99)GFP(+15.99)GAR<br>GSAGPP(+15.99)GATGFP(+15.99)GAAGR<br>Note: The G→N substitution is likely an aberration from the software. It should be an alkylation of the N-terminus by iodoacetamide.
**C0HJP6|CO1A2_MACSX**	216.0	8.49	5	1	Collagen alpha-2(I) chain (Fragment) OS=Macrauchenia	GEAGPAGPAGPAGPR
**C0HJP8|CO1A2_TOXSP**	216.0	8.48	5	1	Collagen alpha-2(I) chain	Further information can be found in the (raw) data files made available via PRIDE (see page S1 for details).

					(Fragment) OS=Toxodon	
**P02465|CO1A2_BOVIN**	216.0	5.65	5	1	Collagen alpha-2(I) chain OS=Bos taurus	
**C0HLH0|CO1A2_BRAVA**	215.9	8.38	5	2	Collagen alpha-2(I) chain (Fragment) OS=Bradypus variegatus	
**C0HLJ4|CO1A2_NOTSH**	215.9	8.13	5	2	Collagen alpha-2(I) chain (Fragment) OS=Nothrotheriops shastensis	
**C0HLI8|CO1A2_SCESW**	213.8	8.5	5	2	Collagen alpha-2(I) chain (Fragment) OS=Scelidodon sp. (strain SLP-2019)	
**C0HJN6|CO1A2_HIPAM**	198.2	6.99	4	1	Collagen alpha-2(I) chain (Fragment) OS=Hippopotamus amphibius	
**C0HJN8|CO1A2_TAPTE**	196.9	6.59	4	1	Collagen alpha-2(I) chain (Fragment) OS=Tapirus terrestris	
**C0HLJ8|CO1A2_MEGJE**	196.3	7.44	4	2	Collagen alpha-2(I) chain (Fragment) OS=Megalonyx jeffersonii	
**C0HLH4|CO1A2_ACRSX**	196.3	7.03	4	2	Collagen alpha-2(I) chain (Fragment) OS=Acratocnus sp. (strain SLP-2019)	
**C0HLI4|CO1A2_GLORB**	196.3	6.49	4	2	Collagen alpha-2(I) chain (Fragment) OS=Glossotherium robustum	
**C0HLG8|CO1A2_CHOHO**	196.3	6.46	4	2	Collagen alpha-2(I) chain (Fragment) OS=Choloe	

					pus hoffmanni	
**C0HLJ0|CO1A2_NEOCO**	196.3	6.43	4	2	Collagen alpha-2(I) chain (Fragment) OS=Neocnus comes	
**C0HJP4|CO1A2_MYLDA**	196.3	6.29	4	2	Collagen alpha-2(I) chain (Fragment) OS=Mylodon darwinii	
**C0HLK0|CO1A2_PARHA**	196.3	6.24	4	2	Collagen alpha-2(I) chain (Fragment) OS=Paramylodon harlani	
**C0HLJ6|CO1A2_MEGAE**	196.3	6.19	4	2	Collagen alpha-2(I) chain (Fragment) OS=Megatherium americanum	
**C0HJN4|CO1A2_ORYAF**	196.3	6.44	4	1	Collagen alpha-2(I) chain (Fragment) OS=Orycteropus afer	
**P08123|CO1A2_HUMAN**	195.4	4.61	4	1	Collagen alpha-2(I) chain OS=Homo sapiens	
**C0HLH6|CO1A2_NEODO**	173.0	4.8	3	1	Collagen alpha-2(I) chain (Fragment) OS=Neocnus dousman	
**P02466|CO1A2_RAT**	171.8	3.43	3	1	Collagen alpha-2(I) chain OS=Rattus norvegicus	
**C0HLJ2|CO1A2_PARSU**	171.4	5.48	3	2	Collagen alpha-2(I) chain (Fragment) OS=Parocnus serus	
**C0HLH2|CO1A2_ACRYE**	171.4	5.27	3	2	Collagen alpha-2(I) chain (Fragment) OS=Acratocnus ye	
**C0HLI0|CO1A2_GLYSX**	167.2	5.79	3	1	Collagen alpha-2(I) chain (Fragment) OS=Glyptodon sp.	

					(strain SLP-2019)	
**C0HLI2|CO1A2_DOESX**	167.2	5.32	3	1	Collagen alpha-2(I) chain (Fragment) OS=Doedicurus sp.	
**P05539|CO2A1_RAT**	161.3	2.82	3	1	Collagen alpha-1(II) chain OS=Rattus norvegicus	
**P28481|CO2A1_MOUSE**	161.3	2.69	3	1	Collagen alpha-1(II) chain OS=Mus musculus	
**Q01149|CO1A2_MOUSE**	152.0	3.72	3	1	Collagen alpha-2(I) chain OS=Mus musculus	
**C0HLI6|CO1A2_SCESX**	140.3	7.75	2	1	Collagen alpha-2(I) chain (Fragment) OS=Scelidotherium sp. (strain SLP-2019)	
**P02460|CO2A1_CHICK**	128.4	3.61	2	1	Collagen alpha-1(II) chain (Fragment) OS=Gallus gallus	
**P02459|CO2A1_BOVIN**	128.4	2.08	2	1	Collagen alpha-1(II) chain OS=Bos taurus OX=9913	
**P02458|CO2A1_HUMAN**	128.4	2.08	2	1	Collagen alpha-1(II) chain OS=Homo sapiens	
**Q6P4Z2|CO2A1_XENTR**	128.4	2.08	2	1	Collagen alpha-1(II) chain OS=Xenopus tropicalis	
**P86290|CO1A2_BRACN**	95.5	50	1	1	Collagen alpha-2(I) chain (Fragment) OS=Brachylophosaurus canadensis	GSNGEP(+15.99)GSAGPP(+15.99)GPAGLR
**C0HJP2|CO1A2_CYCDI**	92.7	2.15	1	1	Collagen alpha-2(I) chain (Fragment) OS=Cyclopes didactylus	

Sample 4 Modern Turkey (*Meleagris gallopavo*)

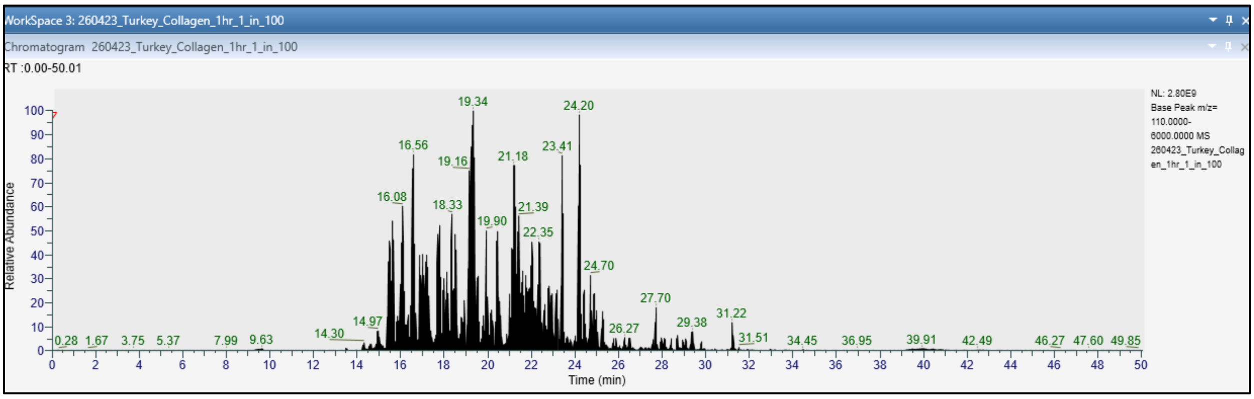

Figure S2 BPI (2.80e9) chromatogram for modern turkey (*Meleagris gallopavo*)

Table S4: Protein match summary for modern turkey (*M. gallopavo*). Database Searched: UniChick (Entries: 43,710).

Accession	Score	Sequence Coverage (%)	No. of Peptides	No. of unique Sequences	Description
**A0A1D5PYU1|A0A1D5PYU1_CHICK**	589.2	81.49	211	159	Collagen type I alpha 1 chain OS=Gallus gallus
**A0A5H1ZRJ7|A0A5H1ZRJ7_CHICK**	567.2	74.69	181	180	Collagen type I alpha 2 chain OS=Gallus gallus
**P02467|CO1A2_CHICK**	567.1	74.69	180	179	Collagen alpha-2(I) chain OS=Gallus gallus
**P02457|CO1A1_CHICK**	535.1	55.33	69	2	Collagen alpha-1(I) chain OS=Gallus gallus
**A0A1L1RPW4|A0A1L1RPW4_CHICK**	530.8	35.68	98	98	Collagen type XII alpha 1 chain OS=Gallus gallus
**P13944|COCA1_CHICK**	521.5	30.35	88	88	Collagen alpha-1(XII) chain OS=Gallus gallus
**A0A8V0YHJ1|A0A8V0YHJ1_CHICK**	451.2	18.94	56	56	Collagen type VI alpha 3 chain OS=Gallus gallus
**P15989|CO6A3_CHICK**	447.5	18.97	55	55	Collagen alpha-3(VI) chain OS=Gallus gallus
**A0A1D5PE57|A0A1D5PE57_CHICK**	441.3	39.45	43	15	Collagen type III alpha 1 chain OS=Gallus gallus
**P12105|CO3A1_CHICK**	378.8	28.53	28	1	Collagen alpha-1(III) chain OS=Gallus gallus
**P15988|CO6A2_CHICK**	357.7	23.29	22	22	Collagen alpha-2(VI) chain OS=Gallus gallus
**A0A8V1A8M8|A0A8V1A8M8_CHICK**	348.5	15.25	21	18	Collagen type V alpha 1 chain OS=Gallus gallus
**A0A8V0YIC4|A0A8V0YIC4_CHICK**	345.4	27.03	19	17	Collagen type V alpha 2 chain OS=Gallus gallus
**P20785|CO6A1_CHICK**	334.5	18.16	18	18	Collagen alpha-1(VI) chain OS=Gallus gallus
**A0A8V0YNN9|A0A8V0YNN9_CHICK**	334.5	16.07	18	18	Collagen type VI alpha 1 chain OS=Gallus gallus
**P02460|CO2A1_CHICK**	319.0	26.31	15	10	Collagen alpha-1(II) chain (Fragment) OS=Gallus gallus
**A0A8V0ZUR6|A0A8V0ZUR6_CHICK**	300.2	6.55	10	7	Collagen type XI alpha 1 chain OS=Gallus gallus

**Q90800|Q90800_CHICK**	52.5	3.7	2	2	Collagen type IX alpha 3 chain OS=Gallus gallus
**P32017|CO9A3_CHICK**	52.5	3.7	2	2	Collagen alpha-3(IX) chain OS=Gallus gallus
**A0A8V0YGK6|A0A8V0YGK6_CHICK**	32.3	1.32	1	1	Collagen, type X, alpha 1 OS=Gallus gallus
**A0A8V1ALZ8|A0A8V1ALZ8_CHICK**	30.2	0.63	1	1	Fibrillar collagen NC1 domain-containing protein OS=Gallus gallus
**A0A8V0Z1M4|A0A8V0Z1M4_CHICK**	27.6	0.73	1	1	Collagen type XVI alpha 1 chain OS=Gallus gallus
**Q7LZR2|CO8A1_CHICK**	26.3	0.94	1	1	Collagen alpha-1(VIII) chain OS=Gallus gallus
**P32018|COEA1_CHICK**	23.1	0.37	1	1	Collagen alpha-1(XIV) chain OS=Gallus gallus
**A0A8V0Y3S3|A0A8V0Y3S3_CHICK**	23.1	0.37	1	1	Collagen type XIV alpha 1 chain OS=Gallus gallus
**A0A8V0YLG2|A0A8V0YLG2_CHICK**	22.2	0.59	1	1	Collagen type IV alpha 5 chain OS=Gallus gallus

Table S5: Protein match summary for modern turkey (M. gallopavo). Database Searched: UniTurkey (Entries: 28,236).

Accession	Score	Sequence Coverage (%)	No. of Peptides	No. of unique Sequences	Description
**G3USK9|G3USK9_MELGA**	479.3	18.61	55	55	Collagen type VI alpha 3 chain OS=Meleagris gallopavo OX=9103 GN=COL6A3 PE=4 SV=2
**A0A803YRZ8|A0A803YRZ8_MELGA**	406.1	33.39	30	30	Collagen type XII alpha 1 chain OS=Meleagris gallopavo OX=9103 PE=4 SV=1
**G3USK7|G3USK7_MELGA**	348.4	20.93	20	19	Collagen type VI alpha 2 chain OS=Meleagris gallopavo OX=9103 GN=COL6A2 PE=4 SV=2
**G3URW1|G3URW1_MELGA**	333.1	27.98	22	22	Collagen type V alpha 2 chain OS=Meleagris gallopavo OX=9103 GN=COL5A2 PE=4 SV=2
**G1N609|G1N609_MELGA**	186.8	11.16	3	3	Fibrillar collagen NC1 domain-containing protein OS=Meleagris gallopavo OX=9103 PE=4 SV=3
**A0A803Y1J6|A0A803Y1J6_MELGA**	175.6	11.26	3	3	Collagen type XI alpha 1 chain OS=Meleagris gallopavo OX=9103 PE=4 SV=1
**G1N4L1|G1N4L1_MELGA**	128.6	4.51	2	2	Fibrillar collagen NC1 domain-containing protein OS=Meleagris gallopavo OX=9103 PE=4 SV=3
**A0A803XL27|A0A803XL27_MELGA**	44.3	6.86	2	2	Collagen IV NC1 domain-containing protein OS=Meleagris gallopavo OX=9103 PE=4 SV=1
**G1N4R3|G1N4R3_MELGA**	39.4	0.74	3	3	Collagen type IV alpha 6 chain OS=Meleagris gallopavo OX=9103 GN=COL4A6 PE=4 SV=3
**G1NIW6|G1NIW6_MELGA**	31.6	1.14	2	1	Collagen type XIV alpha 1 chain OS=Meleagris gallopavo OX=9103 GN=COL14A1 PE=4 SV=3

Sample 5. Bovine tendon collagen (*Bos taurus*, 96%)

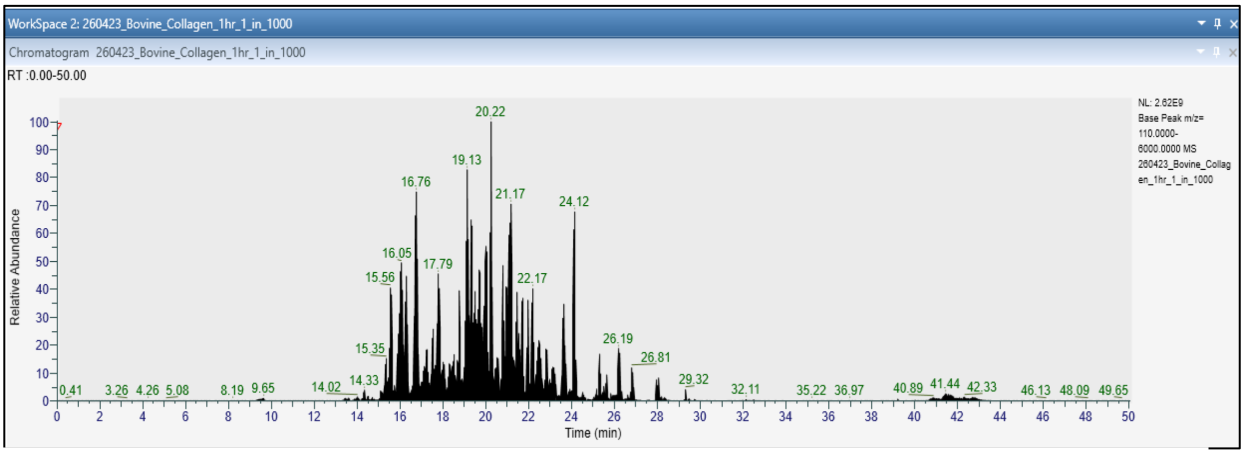

Figure S3 BPI (2.62e9) chromatogram for bovine (*Bos taurus*) tendon collagen (96%)

Table S6: Protein match summary for bovine (*B. taurus*) tendon collagen (96%). Database Searched: UniCow (Entries: 42,151).

Accession	Score	Sequence Coverage (%)	No. of Peptides	No. of unique Peptides	Description
**P02453|CO1A1_BOVIN**	467.2	71.16	141	140	Collagen alpha-1(I) chain OS=Bos taurus
**P02465|CO1A2_BOVIN**	455.7	73.68	125	125	Collagen alpha-2(I) chain OS=Bos taurus
**E1BB91|E1BB91_BOVIN**	376.4	15.64	44	44	Collagen type VI alpha 3 chain OS=Bos taurus
**Q08E14|Q08E14_BOVIN**	365.1	44.41	42	41	Collagen type III alpha 1 chain OS=Bos taurus
**P04258|CO3A1_BOVIN**	356.7	54.81	37	36	Collagen alpha-1(III) chain OS=Bos taurus
**P02459|CO2A1_BOVIN**	329.7	29.39	28	26	Collagen alpha-1(II) chain OS=Bos taurus
**E1BI98|E1BI98_BOVIN**	270.0	16.65	14	14	Collagen type VI alpha 1 chain OS=Bos taurus
**F1MKG2|F1MKG2_BOVIN**	245.3	8.17	9	9	Collagen type VI alpha 2 chain OS=Bos taurus
**A0A3Q1MDT9|A0A3Q1MDT9_BOVIN**	237.3	9.66	9	9	Collagen type V alpha 2 chain OS=Bos taurus
**G3MZI7|G3MZI7_BOVIN**	198.0	3.19	6	5	Collagen type V alpha 1 chain OS=Bos taurus
**F1N401|F1N401_BOVIN**	192.6	1.86	5	5	Collagen type XII alpha 1 chain OS=Bos taurus
**F1N0K0|F1N0K0_BOVIN**	132.0	1.71	2	1	Collagen type XI alpha 1 chain OS=Bos taurus
**Q32S24|COBA2_BOVIN**	131.8	1.96	2	1	Collagen alpha-2(XI) chain OS=Bos taurus
**F1MRP6|F1MRP6_BOVIN**	131.8	1.96	2	1	Collagen type XI alpha 2 chain OS=Bos taurus
**A6QPB3|COHA1_BOVIN**	81.6	0.81	1	1	Collagen alpha-1(XVII) chain OS=Bos taurus
**W0SK50|W0SK50_BOVIN**	81.6	0.81	1	1	Collagen type XVII alpha 1 chain OS=Bos taurus
**A0A3Q1NK51|A0A3Q1NK51_BOVIN**	44.4	0.35	1	1	Collagen type IV alpha 6 chain OS=Bos taurus
**F1MJQ6|F1MJQ6_BOVIN**	37.5	0.86	1	1	Collagen type V alpha 3 chain OS=Bos taurus
**A0A3Q1LXW3|A0A3Q1LXW3_BOVIN**	37.4	0.28	1	1	Collagen type VI alpha 5 chain OS=Bos taurus
**F1N7K7|F1N7K7_BOVIN**	36.7	5.38	1	1	Macrophage receptor with collagenous structure OS=Bos taurus

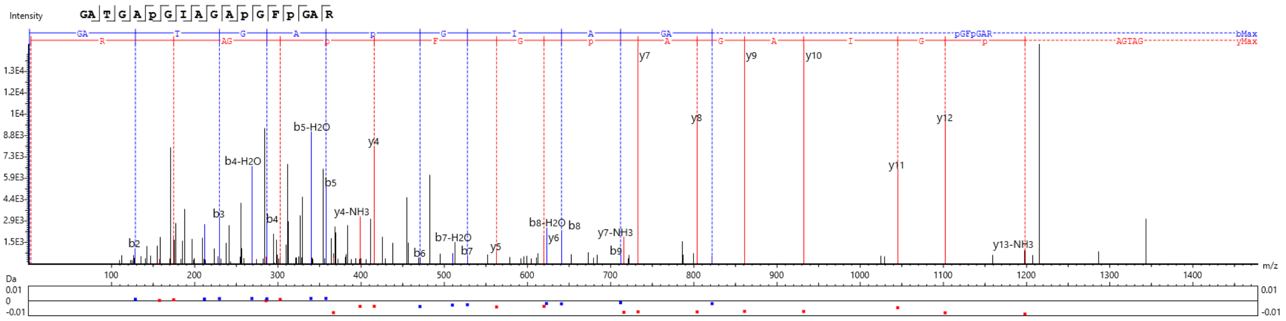
Intensity
GATGApGIAGApGFpGAR

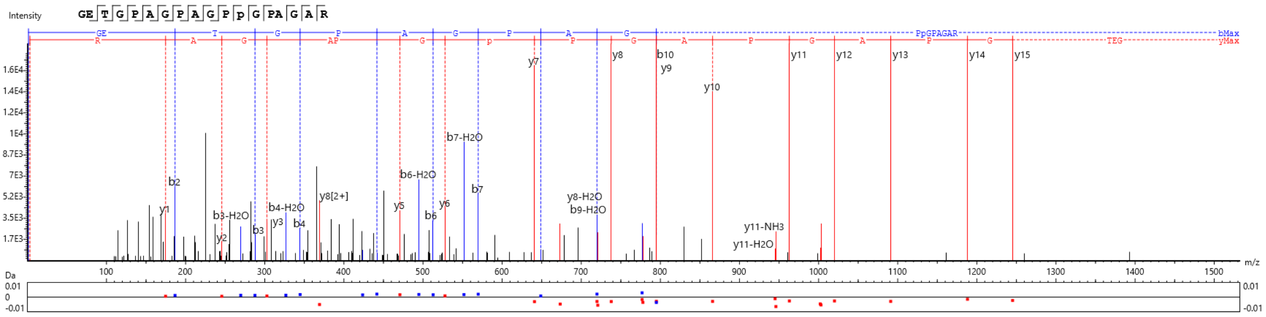
Intensity
GETGPAGPAGPpGPAGAR

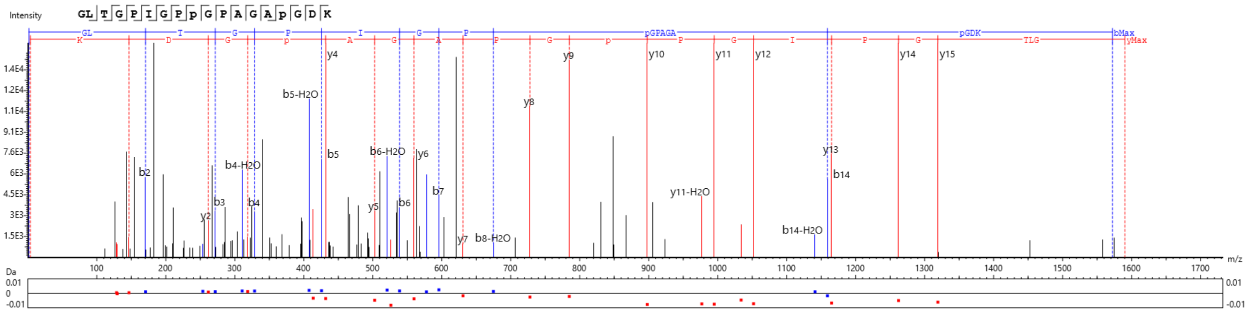
Intensity
GLTGPIGPpGPAGApGDK

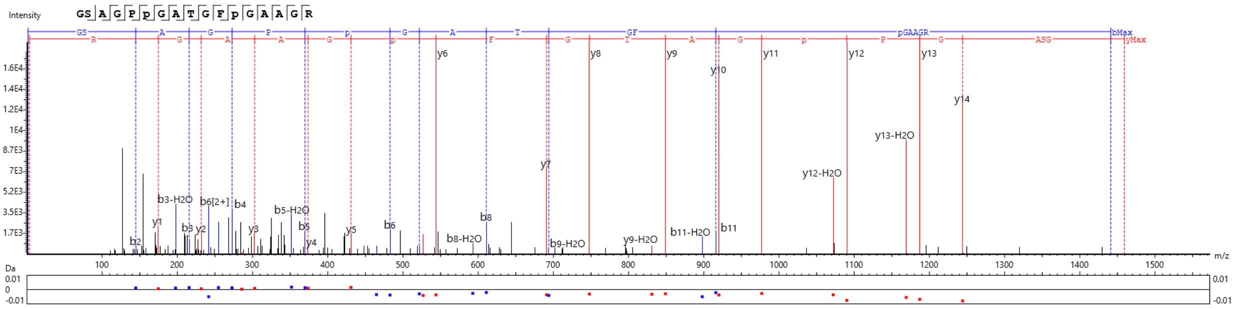
Intensity
GSAGPpGATGFpGAAGR

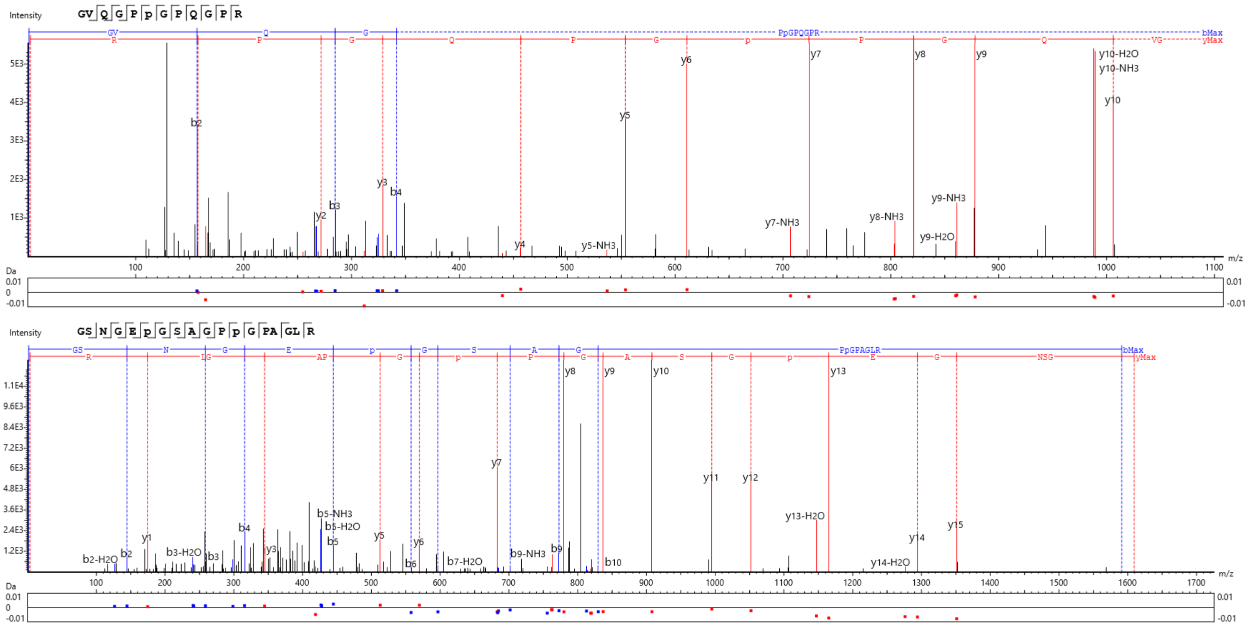

Figure S4 Annotated peptides corresponding to table 1 in the manuscript (and which appear in the same order from top to bottom):

1. GATGAP(+15.99)GIAGAP(+15.99)GFP(+15.99)GAR, *z*=2

2. GETGPAGPAGPP(+15.99)GPAGAR, *z*=2

3. GLTGPIGPP(+15.99)GPAGAP(+15.99)GDK, *z*=2

4. GSAGPP(+15.99)GATGFP(+15.99)GAAGR, *z*=2

5. GVQGPP(+15.99)GPQGPR, *z*=2

6. GSNGEP(+15.99)GSAGPP(+15.99)GPAGLR, *z*=2

Table S7: BLAST of peptide sequences discovered in *Edmontosaurus* UOL GEO.1 (as listed in table S3 above)

Description	Scientific Name	Max Score	Total Score	Query Cover	E value	Per. Ident	Acc. Len	Accession
**GATGAPGIAGAPGFPGAR**								
collagen alpha-1(I) chain isoform X1 [Cygnus olor]	Cygnus olor	54.90	283	100%	7.00E-07	100.00 %	1529	XP_040392463.1
collagen alpha-1(I) chain-like [Engraulis encrasicolus]	Engraulis encrasicolus	54.9	108	100%	7.00E-07	100.00 %	1446	XP_063076789.1
**GETGPAGPAGPPGPAGAR**								
collagen alpha-1(I) chain isoform X1 [Cygnus atratus]	Cygnus atratus	5.540	1303	100%	5.00E-07	100.00 %	1518	XP_035424402.1
RecName: Full=Collagen alpha-1(I) chain; AltName: Full=Alpha-1 type I collagen [Brachylophosaurus canadensis]	Brachylophosaurus canadensis	55.4	176	100%	5.00E-07	100.00 %	113	P86289.1
**GLTGPIGPPGPAGAPGDK**								
COL1A1 and PDGFB fusion transcript [Homo sapiens]	Homo sapiens	56.20	56.2	100%	2.00E-07	100.00 %	62	CAA75876.1
hypothetical protein MJT46_014879 [Ovis ammon polii x Ovis aries]	Ovis ammon polii x Ovis aries	56.20	1153	100%	3.00E-07	100.00 %	1560	KAI4556256.1
**GSAGPPGATGFPGAAGR**								
collagen alpha-1(I) chain [Salvelinus alpinus]	Salvelinus alpinus	51.50	299	100%	9.00E-06	100.00 %	2187	XP_024003453.1
collagen alpha-1(I) chain isoform X1 [Theropithecus gelada]	Theropithecus gelada	51.50	258	100%	9.00E-06	100.00 %	1627	XP_025217417.1
**GVQGPPGPQGPR**								
hypothetical protein GJ496_007221 [Pomphorhynchus laevis]	Pomphorhynchus laevis	40.1	110	100%	0.041	100.00 %	1721	KAI0982436.1
